# Oxidative Addition of C‐F Bonds to the Phosphoranide Ion [P(C_2_F_5_)_2_F_2_]^−^

**DOI:** 10.1002/chem.202503405

**Published:** 2025-12-12

**Authors:** Lukas Hartmann, Beate Neumann, Hans‐Georg Stammler, Mira Kessler, Berthold Hoge

**Affiliations:** ^1^ Fakultät für Chemie, Centrum für Molekulare Materialien Universität Bielefeld Bielefeld Germany; ^2^ Department of Chemistry Henry Bellmon Research Center Oklahoma State University Stilwater USA

**Keywords:** C─F bond activation, phosphate, phosphoranide

## Abstract

Oxidative additions of C─Hal bonds (Hal  =  F, Cl, Br, I) are widely known for a variety of transition metal complexes owing to their range of oxidation states and flexible coordination sphere. Low valent main group element compounds were shown to mimic this reaction behavior, due to the propensity of changing their oxidation state and coordination number by +2. Yet the addition to C─F bonds is especially challenging due to a large bond dissociation energy. While several examples are known for compounds from group 13 and 14, only few reports demonstrate this type of reaction for compounds from group 15. Herein we report the first anionic P(III) species undergoing an oxidative addition of C─F bonds. The tetracoordinated [P(C_2_F_5_)_2_F_2_]^−^‐anion reacts readily with fluorinated arenes, olefins, and acid fluorides yielding respective hexacoordinated P(V) phosphates under mild conditions.

## Graphical abstract

The difluorobis(pentafluoroethyl)phosphoranide anion (λ ^4^ σ ^4^‐phosphate) reacts with substrates containing activated C─F bonds (*R*_f_  =  perfluoroaryl, ‐alkenyl, and ‐acetyl) yielding corresponding trifluorophoshates. The colorless solids exhibit a high thermal stability and are bench stable. This reaction behavior extends the toolbox for possible functionalization of inherently strong C─F bonds.

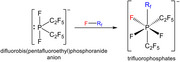

## 1 Introduction

Oxidative additions often represent pivotal steps in catalytic reactions and activations of small molecules such as H_2_, CO_2_, and NH_3_ introducing a simple way for functionalization of unreactive substrates [1, 2]. This mechanistic step, crucial for various catalytic transformations, is well known across several transition metals owing to their multiple stable oxidation states and variable coordination spheres [3, 4]. The oxidative addition step is particularly demanding in the context of C─F bonds due to their inherently high bond dissociation energy [5]. Yet, this obstacle has spurred intensive research in the last decades [4, 6, 7, 8, 9].

More recently, the development of transition‐metal‐free strategies for the activation of otherwise inert bonds has emerged. Amid these efforts, main‐group element compounds offer potential alternatives, although their application has been restricted by limitations in oxidation states and coordination numbers. Nevertheless, low‐valent compounds in low oxidation states, as they are reported for most group 13 [10, 11, 12, 13, 14] and 14 [15, 16, 17, 18, 19, 20] elements, have been proven to undergo oxidative additions.

Examples of oxidative addition reactions involving group 15 compounds remain scarce, particularly in the context of C─F bonds [21, 22, 23, 24, 25, 27]. While phosphenium cations have been documented to undergo oxidative additions to different E─H bonds (E = B, C, Si, N), the activation of C─F bonds has not been elucidated [26, 27]. Reaction of P(CH_3_)_3_ with hexafluoropropene yielding the respective phosphorane, reported by the group of röschenthaler, was the first example of a neutral phosphane undergoing oxidative addition of a C─F bond [23]. Additionally, radosevich and co‐workers reported a geometrically constrained triaminophosphane that underwent oxidative addition to aryl‐C─F bonds, while the group of dobrovetsky demonstrated the same reactivity for a cationic phosphane [24, 25]. Moreover, pang et al. demonstrated the ability of a bismuthinidene to undergo oxidative addition of aryl─C─F bonds [28]. Recently our group reported on the tris(pentafluoroethyl)silanide anion reacting with alkenyl─ and aryl─C─F bonds via oxidative addition [29].

Based on these findings, we aim to extend the oxidative addition reactions from cationic and neutral phosphorus compounds to an anionic P(III) species. The synthesis of the [P(C_2_F_5_)_2_F_2_]^−^ anion as its [EtP_4_H]^+^ salt ([EtP_4_H] = [{(Et_2_N)_3_P = N}_3_P = N(H){C(CH_3_)_3_}]), along with a study of its ligand properties, has recently been reported by our group [30, 31].

## 2 Results and Discussion

The difluorobis(pentafluoroethyl)phosphoranide, [P(C_2_F_5_)_2_F_2_]^−^, reacts with pentafluoropyridine (**a**) as well as hexafluoropropene (**b**), octafluorocyclopentene (**c**), and trifluoroacetylfluoride (**d**) (Scheme 2). Moreover, a reaction with hexafluoropropeneoxide leads to the formation of a carbonylphosphate. These results underscore the versatility of main‐group element compounds in bond activation chemistry.

**SCHEME 1:**
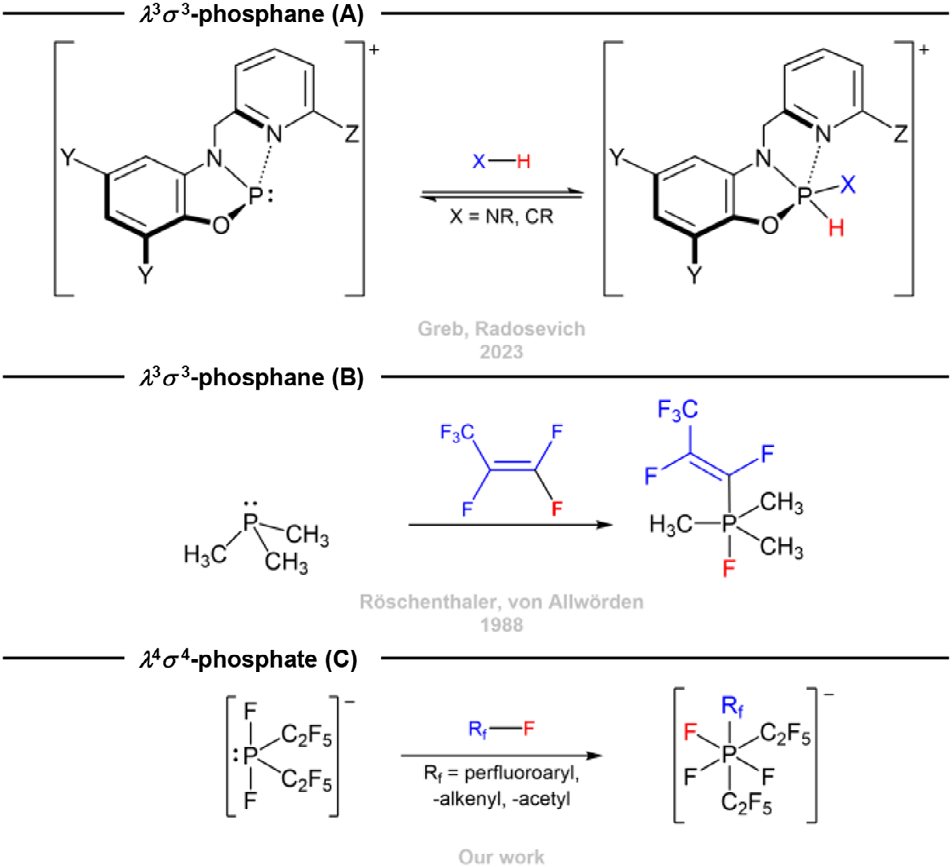
Examples for oxidative addition reactions by a cationic phosphenium (A) [27], a neutral phosphane (B) [23], and an anionic phosphoranide (C).

All reactions exhibit rapid kinetics, achieving completion within hours at room temperature. The respective phosphates were analyzed by multinuclear NMR spectroscopy and single crystal X‐ray diffraction analysis.

**SCHEME 2:**
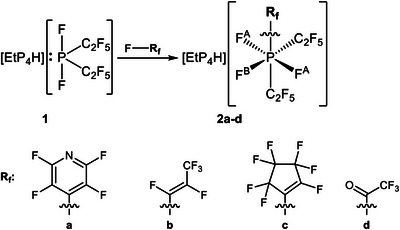
Reaction of phosphoranide 1 with different fluorinated substrates yielding the respective oxidative addition products.

Phosphates **2a‐d** (Scheme 2) give rise to signals between −148 ppm and −152 ppm in the ^31^P NMR spectra, typical for hexavalent P(V) phosphates. A large triplet of doublet splitting caused by coupling between the central phosphorus atom and directly bonded fluorine atoms (F**^A^**, F**^B^**) suggest two types of fluorine atoms. In the ^19^F NMR spectra F^B^ is strongly shifted toward lower field in comparison to F^A^ (Table 1). The CF_2_ units within the C_2_F_5_ groups exhibit two distinct resonances in the ^19^F NMR spectrum, indicating chemical inequivalence of the two groups. Based on these NMR spectroscopic data, a meridional arrangement in solution is suggested, which is consistent with other trifluoroorganylphosphates [32, 33].

**TABLE 1:** ^31^P and ^19^F NMR spectroscopic data of phosphates **2a‐e**.

	*δ*/ppm [P]	*δ*/ppm [PCF_2_CF_3_]	*δ*/ppm [PCF_2_CF_3_]	*δ*/ppm [F_A_] ^1^ *J*_F,P_/Hz	*δ*/ppm [F_B_] ^1^ *J*_F,P_/Hz
2a	−148.7	−117.4	−82.0	−72.0	−24.9
		−117.0	−80.2	892	847
2b	−152.4	−118.5	−82.0	−86.0	−34.5
		−117.1	−80.9	863	842
2c	−151.2	−118.6	−82.0	−77.5	−25.7
		−117.1	−81.1	859	838
2d	−152.2	−117.7	−82.1	−77.1(br)	−47.1
		−117.3	−81.0	897^[^ ^a^ ^]^	906
2e	−151.1	−117.6	−82.1	−89.2	−47.0
		−117.2	−81.0	898	906

^[a]^ Taken from the ^31^P NMR spectrum, as fluorine atoms F^A^ give rise to a broad resonance in the ^19^F NMR spectrum without resolved couplings at room temperature.

**FIGURE 1:**
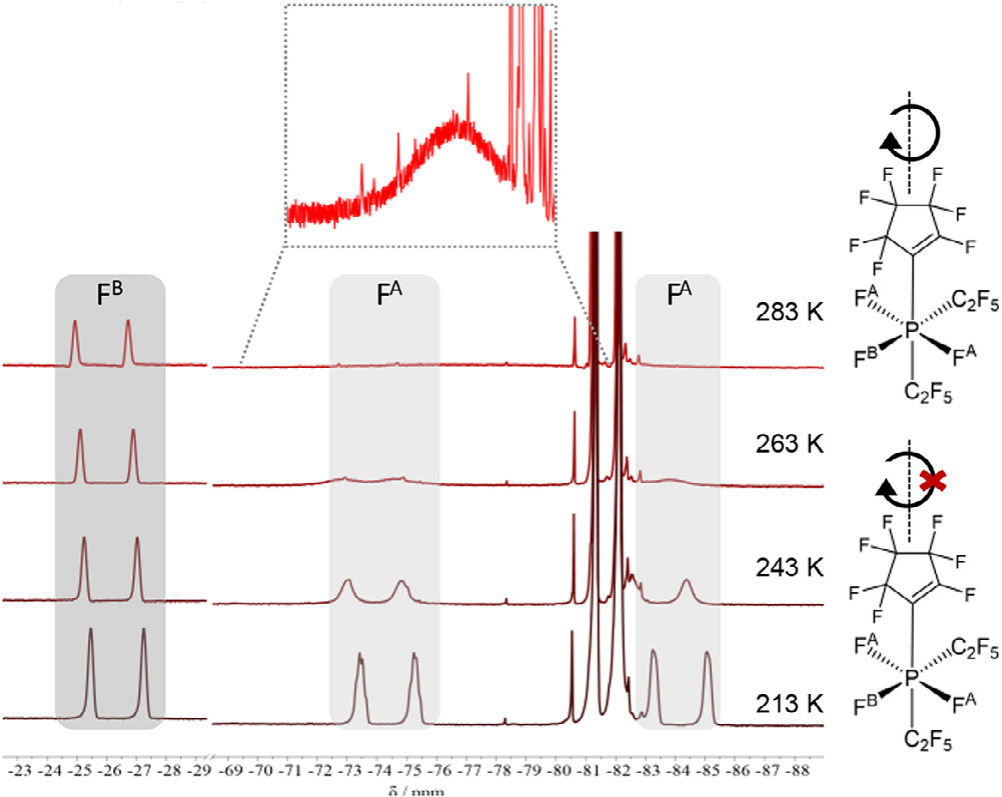
Section from the ^19^F NMR spectrum of phosphate **2c** at different temperatures. Rotation of the cyclopentenyl substituent restricted at low temperatures.

The ^19^F NMR spectroscopic analysis of phosphate **2c** revealed a pronounced temperature dependence. At room temperature, the resonance of fluorine atom B appears as a doublet at −25.7 ppm with a ^1^ *J*_F,P_ coupling constant of 838 Hz, whereas the signal for fluorine atoms A is observed as a single broad resonance at −77.5 ppm without resolved couplings (Figure 1). Upon cooling to 213 K, three distinct doublets emerge (−84.2 ppm, −72.2 ppm, and −25.6 ppm) corresponding to the three fluorine atoms at the phosphorus center. Thus, at low temperatures the rate constants for the exchange processes are decreased sufficiently to resolve the coupling patterns.

**SCHEME 3:**
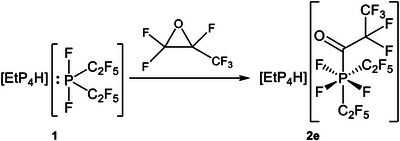
Reaction of phosphoranide 1 with hexafluoropropene oxide yielding phosphate **2e**.

Reaction of phosphoranide **1** with perfluoropropene oxide did not afford the direct oxidative addition product but instead yielded carbonylphosphate **2e** (Scheme 3).

**SCHEME 4:**
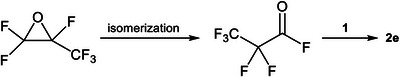
Possible reaction pathway for the reaction of phosphoranide 1 with hexafluoropropene oxide.

A feasible pathway for this transformation may proceed though isomerization of hexafluorenepropene oxide, which has been reported in the literature to occur in the presence of nucleophiles (Scheme 4) [34]. The resulting acid fluoride could then react analogously to trifluoroacetyl fluoride to furnish phosphate **2e**.

Molecular composition of phosphates **2a‐e** was confirmed via high precision elemental analysis and single crystals, suitable for X‐ray diffraction analyses, were obtained for phosphates **2a**,**b**, and **2d**. However, a detailed discussion of the structural parameters of phosphates **2b** and **2d** is not possible due to the use of many restraints (structures depicted in the Supporting Information, Figure S25).

**FIGURE 2:**
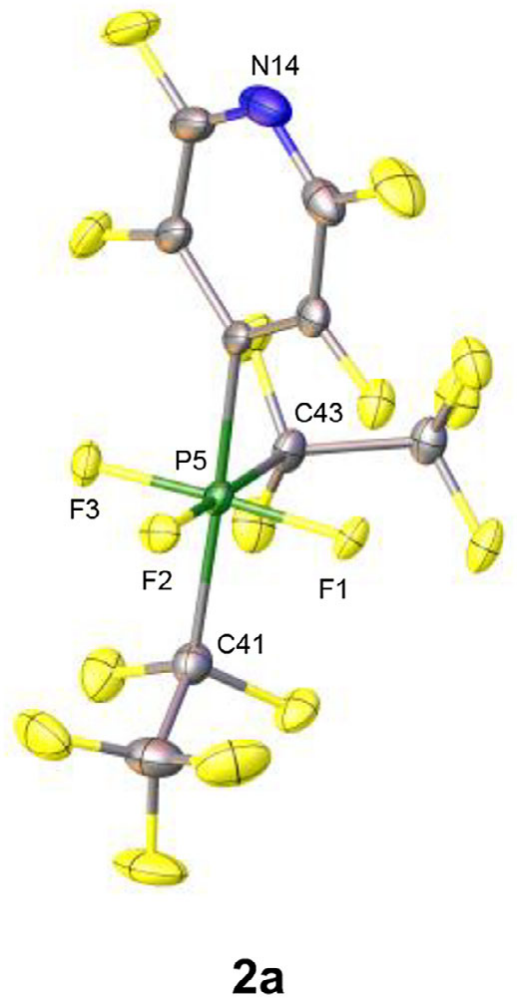
Molecular structure in the solid state of phosphate **2a**. Thermal ellipsoids are shown at the 50% probability level. [EtP_4_H]^+^ cation and minor occupied disordered atoms are omitted for clarity (See Supporting Information for further information). Selected bond lengths and angles are compiled in the Supporting Information Table (S1).

The solid‐state structures of phosphates **2a** (Figure 2), 2**b**, and **2d** reveal central phosphorus atoms adopting a regular octahedral coordination environment [35]. The meridional arrangement, that had already been observed in solution, is retained in the solid state, with the newly introduced substituent positioned trans to one of the C_2_F_5_ groups. The P─F bond lengths in **2a** fall within a rather narrow range of 161.1(2)–161.7(2) pm, which is notably shorter than the P─F bonds in phosphoranide **1** (183.6(5) pm and 173.2(6) pm), in line with the expectations [30]. Remaining structural parameters do not show noteworthy deviations and will therefore not be discussed in detail.

## 3 Conclusion

In summary, we have demonstrated that the difluorobis(pentafluoroethyl)phosphoranide [P(C_2_F_5_)_2_F_2_]^−^ undergoes oxidative addition to C─F bonds, thus establishing the first anionic P(III) species capable of this transformation. The resulting hexacoordinated P(V) phosphates are bench‐stable, colorless solids and were characterized by multinuclear NMR spectroscopy. The molecular composition was confirmed by elemental analysis, whereas single‐crystal X‐ray diffraction confirmed the molecular structures of phosphates **2a**, **b**, and **2d**. This reactivity highlights the versatility of main‐group element compounds and expands their applicability in the activation of strong C─F bonds.

## Conflicts of Interest

The authors declare no conflict of interest.

## Supporting information




**Supporting Information file 1**: Additional references cited within the Supporting Information [36, 37, 38]

**Supporting Information file 2**: chem70542‐sup‐0002‐DataFile.zip

## Data Availability

The data that support the findings of this study are available in the supplementary material of this article.
