# Supplementary material for: Oxidative Addition of C‐F Bonds to the Phosphoranide Ion [P(C2F5)2F2]−
Source: Chemistry. 2025 Dec 12;32(2):e03405. doi: 10.1002/chem.202503405 (PMC12790317; doi:10.1002/chem.202503405)
Supplement: Supplementary file 1 — Supporting Information file 1: Additional references cited within the Supporting Information [36, 37, 38] [file CHEM-32-e03405-s002.docx]

**Supporting Information**

**Oxidative Addition of C-F Bonds to the Phosphoranide Ion
[P(C_2_F_5_)_2_F_2_]^−^**

L. Hartmann, B. Neumann, H.-G. Stammler, M. Kessler*, B.Hoge*

**Table of Contents**

[**1. Experimental Procedures** 2](#_Toc215831750)

[**1.1 General** 2](#_Toc215831751)

[**1.2 Syntheses and NMR spectra** 3](#_Toc215831752)

[**1.2.1** [EtP_4_H][P(C_2_F_5_)_2_(C_5_NF_4_)F_3_] (**2a**) 3](#_Toc215831753)

[**1.2.2** [EtP_4_H][P(C_2_F_5_)_2_(C_3_F_5_)F_3_] (**2b**) 6](#_Toc215831754)

[**1.2.3** [EtP_4_H][P(C_2_F_5_)_2_(C_5_F_7_)F_3_] (**2c**) 8](#_Toc215831755)

[**1.2.4** [EtP_4_H][P(C_2_F_5_)_2_(C_2_F_3_O)F_3_] (**2d**) 11](#_Toc215831756)

[**1.2.5** [EtP_4_H][P(C_2_F_5_)_2_(C_3_F_5_O)F_3_] (**2e**) 13](#_Toc215831757)

[**2. X-ray Data** 16](#_Toc215831758)

[**3. References** 0](#_Toc215831759)

# **1. Experimental Procedures**

**1.1 General**

All chemicals were purchased from commercial sources and used without further purification and solvents were dried according to standard procedures. [EtP_4_H][P(C_2_F_5_)_2_F_2_] was prepared as described in the literature.^[30]^ All operations were conducted using a standard high vacuum system. Handling of nonvolatile compounds were performed under normal Schlenk conditions using dry nitrogen as an inert atmosphere. NMR spectra were recorded on a Bruker *Avance 500*, *500HD* or *NEO 600* using the indicated deuterated solvent. Positive shifts are downfield from the external standards Si(CH_3_)_4_ (^1^H, ^13^C), CF_3_Cl (^19^F) and H_3_PO_4_ (^31^P). IR spectroscopic measurements were performed on a *Bruker* *Alpha-FT-IR spectrometer* equipped with a diamond crystal. ESI mass spectra were recorded on a *ZQ2000* single quadrupole mass spectrometer (Waters, Manchester, UK) equipped with an ESI source, operating with a spray voltage of 3.5 kV. Elemental analyses were performed by *Mikroanalytisches Laboratorium Kolbe* (Oberhausen, Germany). Melting points were measured on a Mettler Toledo Mp70 Melting Point System. Single-crystal X-ray analyses were performed on a *Rigaku Supernova diffractometer* using Mo-K*α* ($\lambda$ = 0.71073 Å) or Cu-K*α* ($\lambda$ = 1.54184 Å) radiation. Crystals were kept at 100.0(1) K during data collection. Using Olex2, the structure was solved with SHELXT structure solution program using intrinsic phasing and refined with olex2.refine or SHELXL refinement package Least square minimization.

The ^1^H NMR spectrum of all compounds shows the same signals and is therefore only depicted for phosphate **2a.**

**1.2 Syntheses and NMR spectra**

### **1.2.1** [EtP_4_H][P(C_2_F_5_)_2_(C_5_NF_4_)F_3_] (**2a**)

Bromobis(pentafluoroethyl)phosphane (323 mg, 0.93 mmol) and Et_2_O (20 mL) were condensed on AgF (461 mg, 3.63 mmol) and stirred for 45 min after thawing. The black suspension was directly filtered on [EtP_4_H]Cl (694 mg, 0.75 mmol). The reaction mixture was stirred for 20 min and C_5_F_5_N (165 mg, 0.98 mmol) was added. After 16 h the grey suspension was filtered and all volatile components were removed. Single crystals suitable for X-Ray diffraction were obtained from a concentrated MeCN solution at −30 °C. The remaining crystals were washed with *n*-hexane (2 x 8 mL) and the product was obtained as a colorless solid (139 mg, 103 μmol, 14%).

**2a**: Cation omitted for clarity.

**^1^H NMR** (CDCl_3_, 303 K, 500 MHz): δ / ppm = 1.1 (t, ^3^*J*_H,H_ = 7 Hz, 54 H, CH_2_C**H_3_**), 1.3 (s, 9 H, C(C**H_3_**)_3_), 2.0 (d, ^2^*J*_H,P_ = 7 Hz, 1 H, N**H**), 3.1 (dquar, ^3^*J*_H,P_ = 10 Hz, ^3^*J*_H,H_ = 7 Hz, 36 H, C**H_2_**).

**^13^C{^1^H} NMR** (CDCl_3_, 303 K, 126 MHz): δ / ppm = 13.5 (d, ^3^*J*_C,P_ = 4 Hz, 6 C, CH_2_**C**H_3_), 31.5 (d, ^3^*J*_C,P_ = 5 Hz, 3 C, C(**C**H_3_)_3_), 39.2 (d, ^2^*J*_C,P_ = 5 Hz, 6 C, **C**H_2_), 50.8 (d, ^2^*J*_C,P_ = 4 Hz, 1 C, **C**(CH_3_)_3_).

**^13^C{^19^F} NMR** (CDCl_3_, 303 K, 150 MHz, O2P = −80.0 ppm): δ / ppm = 120.7 (d, ^2^*J*_C,P_ = 24 Hz, 1 C, **C**F_3_), 120.9 (d, ^3^*J*_C,P_ = 25 Hz, 1 C, **C**F_3_), 144.1 (s, 1 C, **C^2^**), 144.2 (m, 1 C, **C^1^**).

**^13^C{^19^F} NMR** (CDCl_3_, 303 K, 150 MHz, O2P = −130.0 ppm): δ / ppm = 118.3 (s, 1 C, **C**F_2_), 119.5 (s, 1 C, **C**F_2_), 142.4 (s, 1 C, **C^1^**), 144.2 (d, ^2^*J*_C,P_ = 16 Hz, 1 C, **C^2^**).

No signals observed for C^3^.

**^19^F NMR** (CDCl_3_, 303 K, 470 MHz): δ / ppm = −130.5 (m, 2 F, C^1^**F**), −117.4 (dm, ^2^*J*_F,P_ = 89 Hz, 2 F, C**F_2_**), −117.0 (dm, ^2^*J*_F,P_ = 95 Hz, 2 F, C**F_2_**), −96.0 (m, 2 F, C^2^**F**), −82.0 (m, 3 F, C**F_3_**), −80.2 (m, 3 F, C**F_3_**), −72.0 (dm, ^1^*J*_F,P_ = 892 Hz, 2 F, P**F_2_**), −24.9 (dm, ^1^*J*_F,P_ = 847 Hz, 1 F, P**F**).

**^31^P NMR** (CDCl_3_, 303 K, 202 MHz): δ / ppm = −148.7 (tdtt, ^1^*J*_F,P_ = 892 Hz, ^1^*J*_F,P_ = 847 Hz, ^2^*J*_F,P_ = 95 Hz, ^2^*J*_F,P_ = 89 Hz, 1 P, **P**F_3_), −33.8 (quartd, ^2^*J*_P,P_ = 70 Hz, ^2^*J*_H,P_ = 7 Hz, 1 P, **P**(NH*^t^*Bu)), 7.5 (dtridec, ^2^*J*_P,P_ = 70 Hz, ^2^*J*_H,P_ = 10 Hz, 3 P, **P**(NEt_2_)_3_).

**MS (ESI, pos., MeCN)**, *m/z* (%): 886 [EtP_4_H]^+^ (100).

**MS (ESI, neg., MeCN)**, *m/z* (%): 326 (28) [PF_3_(C_2_F_5_)_2_H]^−^, 475 (100) [PF_3_(C_2_F_5_)_2_(C_5_NF_4_)]^−^.

**IR (ATR, solid)**: *ṽ* (cm^−1^): 1426 (w), 1378 (w), 1351 (m), 1280 (m), 1234 (w), 1203 (s), 1173 (vs), 1133 (m), 1102 (w), 1073 (w), 1055 (w), 1018 (s), 943 (s), 834 (m), 793 (m), 757 (w), 719 (m), 701 (s), 611 (s), 575 (w), 507 (m), 459 (s), 438 (m).

**Elemental analysis**: Calcd. (%) C (43.17) H (7.39) N (14.38) F (23.69) P (11.36) Found (%) C (43.27) H (7.31) N (14.31) F (23.58) P (11.38).


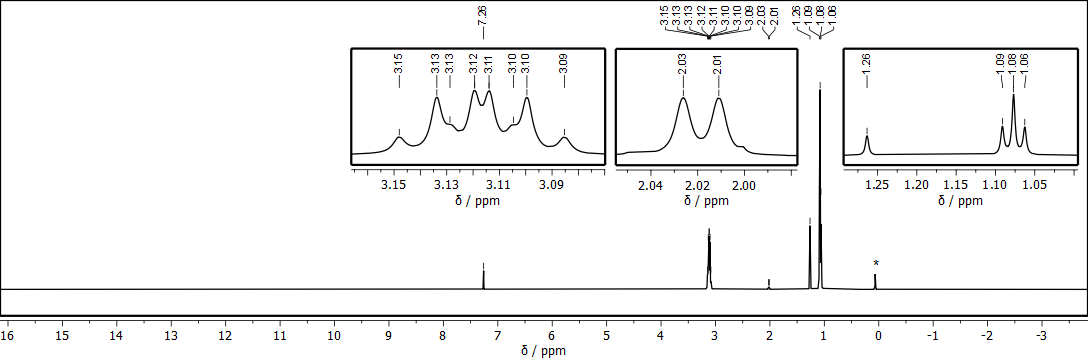


**Figure S 1.** ^1^H NMR spectrum of [EtP_4_H][P(C_2_F_5_)_2_(C_5_NF_4_)F_3_] in CDCl_3_. Solvent signal 7.26 ppm. *silicon grease.


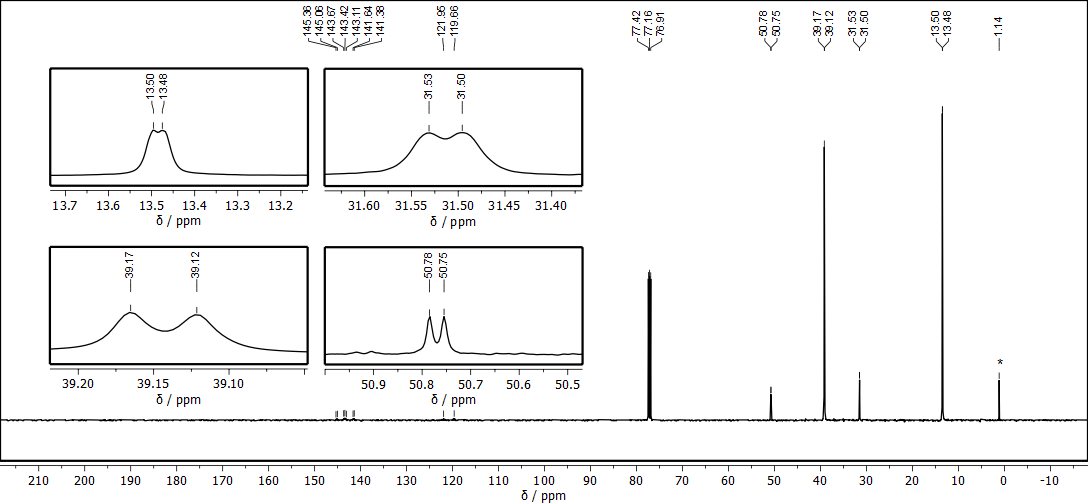


**Figure S 2.** ^13^C{^1^H} NMR spectrum of [EtP_4_H][P(C_2_F_5_)_2_(C_5_NF_4_)F_3_] in CDCl_3_. Solvent signal 77.16 ppm. *silicon grease.


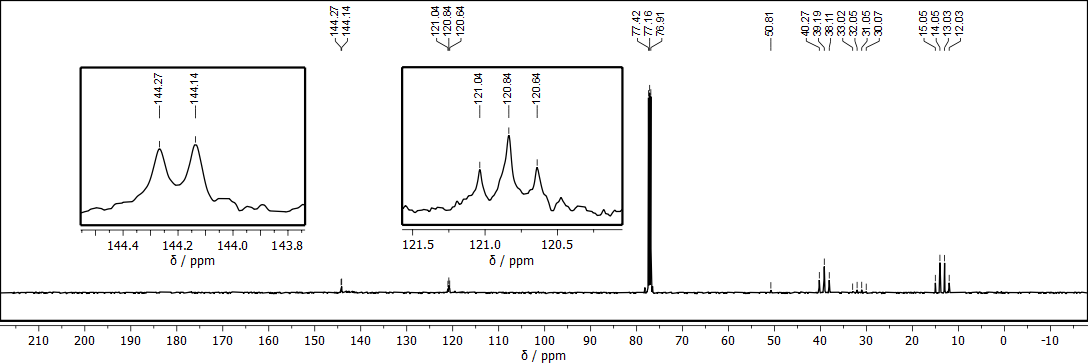


**Figure S 3.** ^13^C{^19^F} NMR spectrum of [EtP_4_H][P(C_2_F_5_)_2_(C_5_NF_4_)F_3_] in CDCl_3_ (O2P = −80.0 ppm). Solvent signal 77.16 ppm.


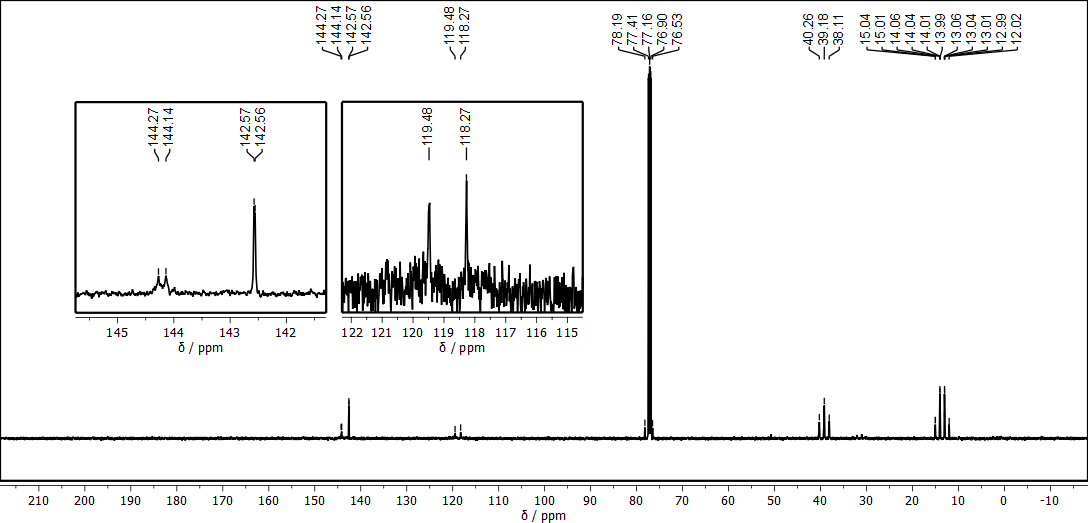


**Figure S 4.** ^13^C{^19^F} NMR spectrum of [EtP_4_H][P(C_2_F_5_)_2_(C_5_NF_4_)F_3_] in CDCl_3_ (O2P = −130.0 ppm). Solvent signal 77.16 ppm.


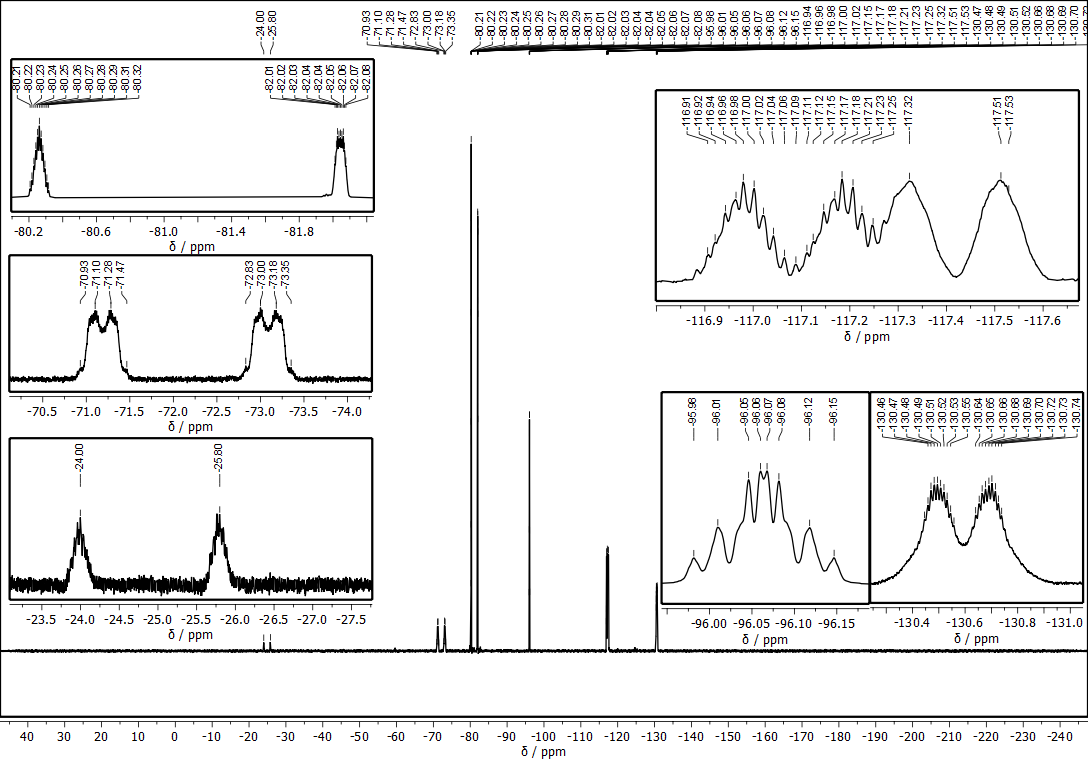


**Figure S 5.** ^19^F NMR spectrum of [EtP_4_H][P(C_2_F_5_)_2_(C_5_NF_4_)F_3_] in CDCl_3_.


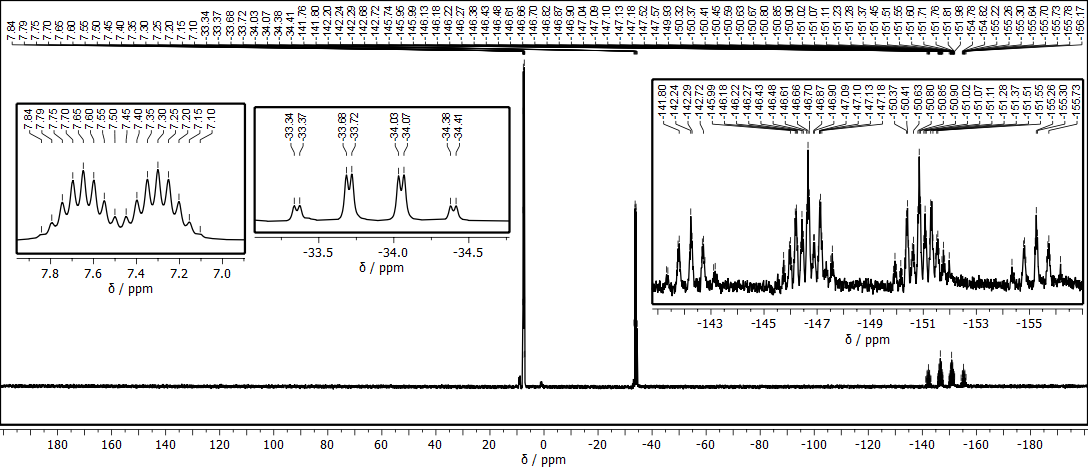


**Figure S 6.** ^31^P NMR spectrum of [EtP_4_H][P(C_2_F_5_)_2_(C_5_NF_4_)F_3_] in CDCl_3_

### **1.2.2** [EtP_4_H][P(C_2_F_5_)_2_(C_3_F_5_)F_3_] (**2b**)

Bromobis(pentafluoroethyl)phosphane (366 mg, 1.05 mmol) and Et_2_O (30 mL) were condensed on AgF (392 mg, 3.09 mmol) and stirred for 45 min after thawing. The black suspension was directly filtered on [EtP_4_H]Cl (856 mg, 0.93 mmol). The reaction mixture was stirred for another 45 min and C_3_F_6_ (2.5 mmol) was condensed onto it. After 16 h the grey suspension was filtered and all volatile components were removed. Crystals suitable for X-Ray diffraction were obtained from a concentrated MeCN solution at −30 °C. Remaining crystals were washed with *n*-pentane (2 x 8 mL) and the product was obtained as a colorless solid (367 mg, 0.27 mmol, 42%).

**2b**: Cation omitted for clarity.

**^1^H NMR** (CDCl_3_, 303 K, 500 MHz): δ / ppm = 1.0 (t, ^3^*J*_H,H_ = 7 Hz, 54 H, CH_2_C**H_3_**), 1.2 (s, 9 H, C(C**H_3_**)_3_), 1.9 (d, ^2^*J*_H,P_ = 7 Hz, 1 H, N**H**), 3.0 (dquar, ^3^*J*_H,P_ = 10 Hz, ^3^*J*_H,H_ = 7 Hz, 36 H, C**H_2_**).

**^13^C{^1^H} NMR** (CDCl_3_, 303 K, 126 MHz): δ / ppm = 12.5 (d, ^3^*J*_C,P_ = 4 Hz, 6 C, CH_2_**C**H_3_), 30.5 (d, ^3^*J*_C,P_ = 5 Hz, 3 C, C(**C**H_3_)_3_), 38.1 (d, ^2^*J*_C,P_ = 5 Hz, 6 C, **C**H_2_), 49.8 (d, ^2^*J*_C,P_ = 4 Hz, 1 C, **C**(CH_3_)_3_).

**^13^C{^19^F} NMR** (CDCl_3_, 303 K, 126 MHz, O2P = −76.0 ppm): δ / ppm = 116.2-120.1 (m, 5 C, overlapping CF_3_ and CF_2_), 140.0 (m, 1 C, PC**C**F), 162.0 (m, 1 C, P**C**F).

Signals caused by CF_3_- and CF_2_- groups could not be distinguished by ^13^C{^19^F}-NMR experiments.

**^19^F NMR** (CDCl_3_, 303 K, 470 MHz): δ / ppm = −166.2 (ddm, ^3^*J*_F,F_ = 132 Hz, ^3^*J*_F,P_ = 9 Hz, 1 F, PCC**F**), −144.9 (ddm, ^3^*J*_F,F_ = 132 Hz, ^2^*J*_F,P_ = 96 Hz, 1 F, PC**F**), −118.5 (dm, ^2^*J*_F,P_ = 88 Hz, 2 F, C**F_2_**), −117.1 (dm, ^2^*J*_F,P_ = 99 Hz, 2 F, C**F_2_**), −86.0 (dm, ^1^*J*_F,P_ = 863 Hz, 2 F, P**F_2_**), −82.0 (m, 3 F, CF_2_C**F_3_**), −80.9 (m, 3 F, CF_2_C**F_3_**), −67.1 (dd, ^3^*J*_F,F_ = 23 Hz, ^4^*J*_F,F_ = 10 Hz, 2 F, PCCC**F_3_**), −34.5 (dm, ^1^*J*_F,P_ = 842 Hz, P**F**).

**^31^P NMR** (CDCl_3_, 303 K, 202 MHz): δ / ppm = −152.4 (tdtdtd, ^1^*J*_F,P_ = 863 Hz, ^1^*J*_F,P_ = 842 Hz, ^2^*J*_F,P_ = 99 Hz, ^2^*J*_F,P_ = 96 Hz, ^2^*J*_F,P_ = 88 Hz, ^3^*J*_F,P_ = 9 Hz, 1 P, **P**F_3_), −33.8 (quartd, ^2^*J*_P,P_ = 70 Hz, ^2^*J*_H,P_ = 7 Hz, 1 P, **P**(NH*^t^*Bu)), 7.5 (dtridec, ^2^*J*_P,P_ = 70 Hz, ^2^*J*_H,P_ = 10 Hz, 3 P, **P**(NEt_2_)_3_).

**IR (ATR, solid)**: *ṽ* (cm^−1^): 2972 (w), 2937 (w), 2874 (w), 1465 (w), 1379 (m), 1355 (m), 1285 (s), 1200 (s), 1175 (s), 1103 (m), 1070 (m), 1055 (m), 1019 (s), 848 (w), 809 (m), 794 (s), 757 (m), 724 (m), 701 (s), 612 (s), 529 (s), 509 (s), 446 (s), 418 (w).

**Elemental analysis**: Calcd. (%) C (42.00) H (7.50) N (13.55) F (25.44) P (11.52); Found (%) C (41.82) H (7.54) N (13.51) F (25.39) P (11.47).

**M.p**.: 162 °C.


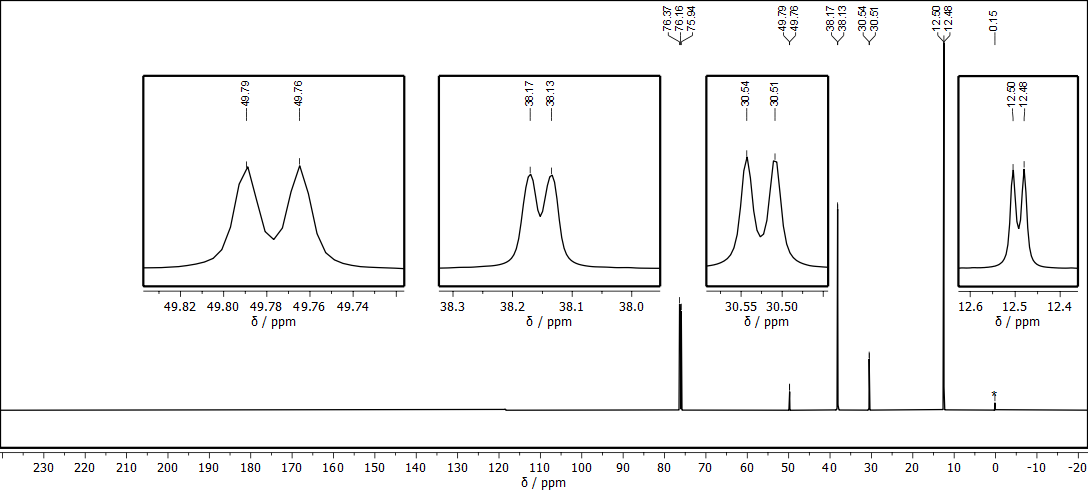


**Figure S 7.** ^13^C{^1^H} NMR spectrum of [EtP_4_H][P(C_2_F_5_)_2_(C_3_F_5_)F_3_] in CDCl_3_. Solvent signal 77.16 ppm. *silicon grease.


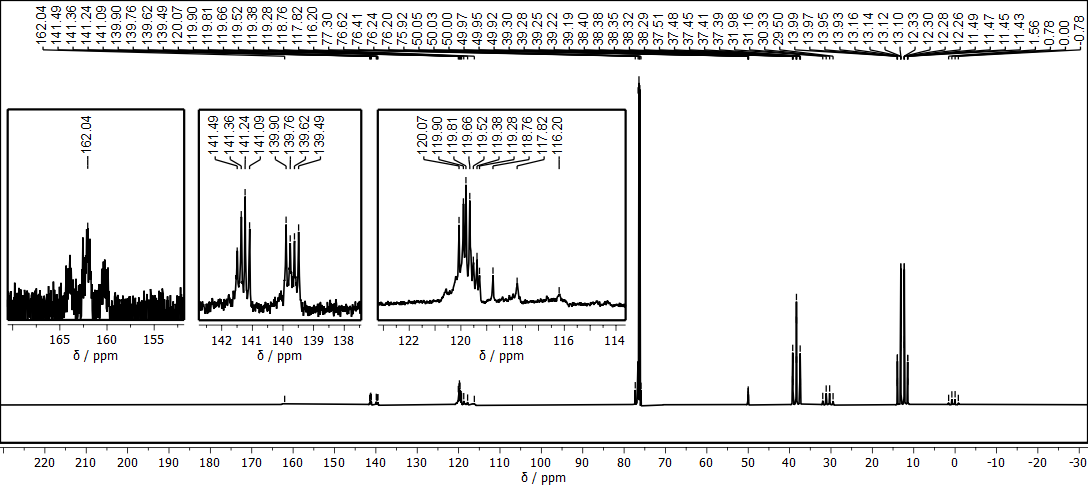


**Figure S 8.** ^13^C{^19^F} NMR spectrum of [EtP_4_H][P(C_2_F_5_)_2_(C_3_F_5_)F_3_] in CDCl_3_ (O2P = −76.0 ppm). Solvent signal 77.16 ppm.


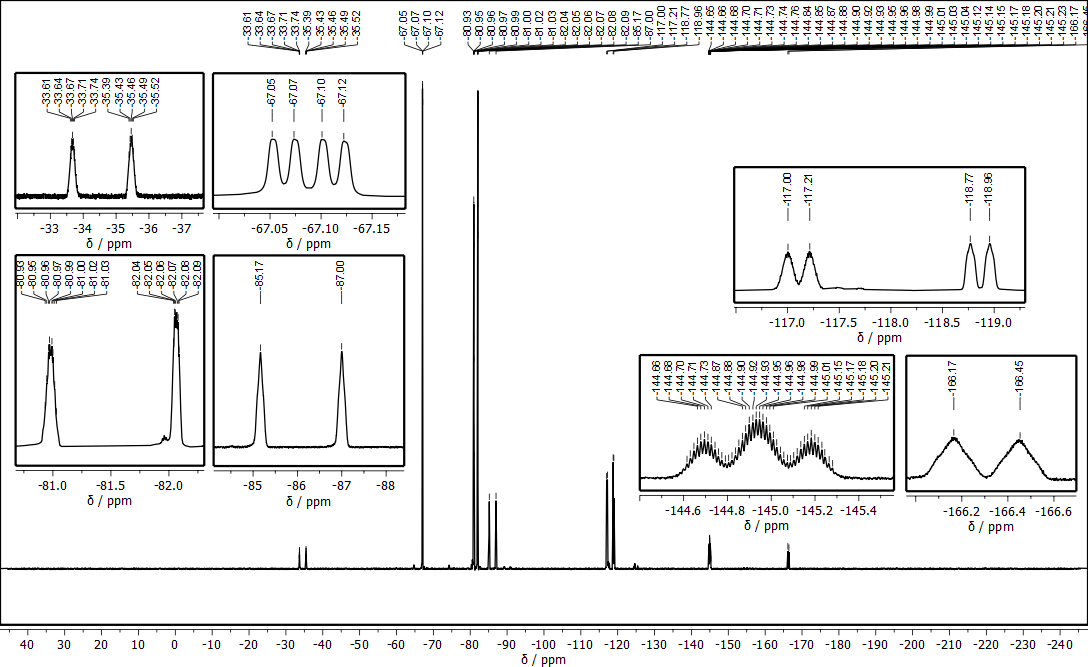


**Figure S 9.** ^19^F NMR spectrum of [EtP_4_H][P(C_2_F_5_)_2_(C_3_F_5_)F_3_] in CDCl_3_.


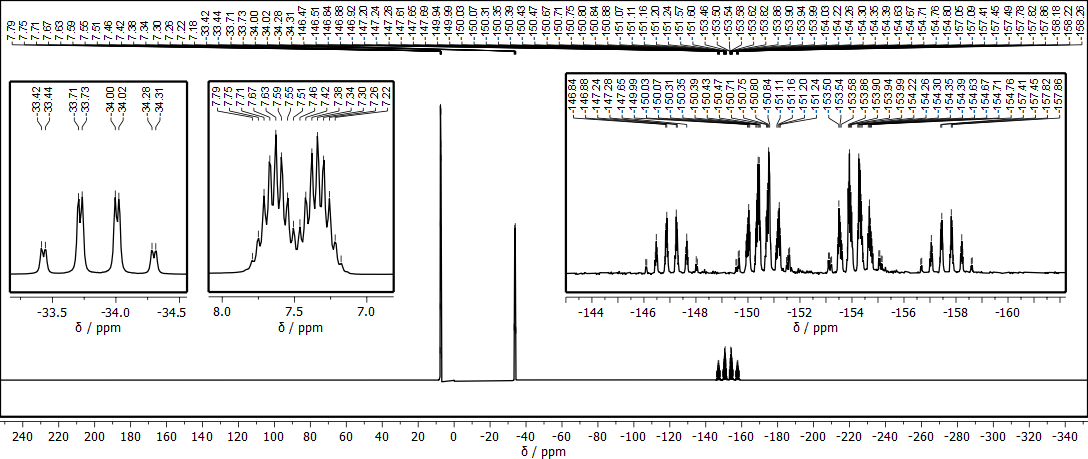


**Figure S 10.** ^31^P NMR spectrum of [EtP_4_H][P(C_2_F_5_)_2_(C_3_F_5_)F_3_] in CDCl_3_.


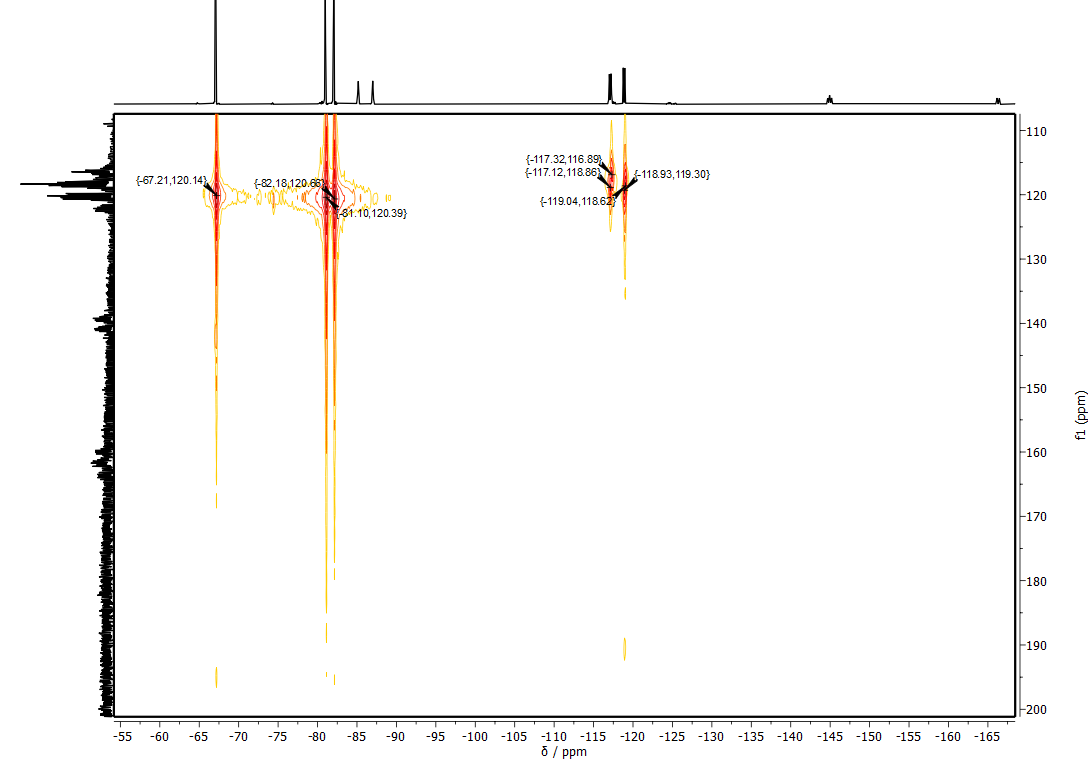


**Figure S 11.** ^19^F^13^C-HMQC NMR spectrum of [EtP_4_H][P(C_2_F_5_)_2_(C_3_F_5_)F_3_] in CDCl_3_ (O1P = −75.0 ppm).

### **1.2.3** [EtP_4_H][P(C_2_F_5_)_2_(C_5_F_7_)F_3_] (**2c**)

Bromobis(pentafluoroethyl)phosphane (350 mg, 1.00 mmol) and Et_2_O (20 mL) were condensed on AgF (754 mg, 5.94 mmol) and stirred for 45 min after thawing. The black suspension was directly filtered on [EtP_4_H]Cl (746 mg, 0.81 mmol). The reaction mixture was stirred for 20 min and C_5_F_8_ (274 mg, 1.29 mmol) was added. After 1 d the grey suspension was filtered and all volatile components were removed. Crystallization from a concentrated MeCN-solution at −30°C afforded crystals, which were washed with *n*‑pentane (2 x 7 mL) and the product was obtained as a colorless solid (483 mg, 0.34 mmol, 42%).

**2c**: Cation omitted for clarity.

**^1^H NMR** (CDCl_3_, 303 K, 500 MHz): δ / ppm = 1.1 (t, ^3^*J*_H,H_ = 7 Hz, 54 H, CH_2_C**H_3_**), 1.3 (s, 9 H, C(C**H_3_**)_3_), 2.1 (d, ^2^*J*_H,P_ = 7 Hz, 1 H, N**H**), 3.1 (dquar, ^3^*J*_H,P_ = 10 Hz, ^3^*J*_H,H_ = 7 Hz, 36 H, C**H_2_**).

**^13^C{^1^H} NMR** (CDCl_3_, 303 K, 126 MHz): δ / ppm = 13.5 (d, ^3^*J*_C,P_ = 4 Hz, 6 C, CH_2_**C**H_3_), 31.5 (d, ^3^*J*_C,P_ = 5 Hz, 3 C, C(**C**H_3_)_3_), 39.2 (d, ^2^*J*_C,P_ = 5 Hz, 6 C, **C**H_2_), 50.8 (d, ^2^*J*_C,P_ = 4 Hz, 1 C, **C**(CH_3_)_3_).

**^13^C{^19^F} NMR** (CDCl_3_, 303 K, 126 MHz, O2P = −80.0 ppm): δ / ppm = 116.9 (s, 1 C, **C**^4^), 120.6 (d, ^2^*J*_C,P_ = 24 Hz, 1 C, C**F_3_**), 120.8 (d, ^2^*J*_C,P_ = 28 Hz, C**F_3_**), 130.8 (s, 1 C, **C**^5^), 153.6 (s, 1 C, **C^1^**).

**^13^C{^19^F} NMR** (CDCl_3_, 303 K, 126 MHz, O2P = −130.0 ppm): δ / ppm = 111.7 (d, ^3^*J*_C,P_ = 16 Hz, 1 C, **C**^3^), 110.9 (d, ^3^*J*_C,P_ = 11 Hz, 1 C, **C^2^**)

No resonances observed for CF_2_-groups within the C_2_F_5_ units.

**^19^F NMR** (CDCl_3_, 303 K, 470 MHz): δ / ppm = −131.2 (m, 2 F, C^3^**F_2_**), −120.1 (m, 3 F, C^1^**F**, and C^2^**F_2_**), −118.6 (m, 2 F, PC**F_2_**CF_3_), −117.0 (dm, ^2^*J*_F,P_ = 97 Hz, 2 F, PC**F_2_**CF_3_), −105.8 (m, 2 F, C^4^**F_2_**), −82.0 (m, 3 F, C**F_3_**), −81.1 (m, 3 F, C**F_3_**), −77.5 (dm(br), ^1^*J*_F,P_ = 859 Hz, 2 F, P**F_2_**), −25.7 (dm, ^1^*J*_F,P_ = 838 Hz, 1 F, P**F**).

**^31^P NMR** (CDCl_3_, 303 K, 202 MHz): δ / ppm = −151.2 (tdtt, ^1^*J*_F,P_ = 855 Hz, ^1^*J*_F,P_ = 838 Hz, ^2^*J*_F,P_ = 97, ^2^*J*_F,P_ = 97, 1 P, **P**F_3_)), ­−33.8 (quartd, ^2^*J*_P,P_ = 70 Hz, ^2^*J*_H,P_ = 7 Hz, 1 P, **P**(NH*^t^*Bu)), 7.5 (dtridec, ^2^*J*_P,P_ = 70 Hz, ^2^*J*_H,P_ = 10 Hz, 3 P, **P**(NEt_2_)_3_).

**MS (ESI, pos., MeCN)**, *m/z* (%): 886 [EtP_4_H]^+^ (100).

**MS (ESI, neg., MeCN)**, *m/z* (%): 327 [PF_3_(C_2_F_5_)_2_H]^-^, 519 [PF_3_(C_2_F_5_)_2_(C_5_F_7_)]^−^.

**IR (ATR, solid)**: *ṽ* (cm^-1^): 2975 (w), 2937 (w), 2874 (w), 1666 (w), 1377 (w), 1351 (w), 1279 (m), 1245 (m), 1204 (s), 1174 (vs), 1137 (s), 1103 (m), 1054 (m), 1018 (s), 971 (s), 942 (s), 843 (w), 813 (m), 794 (m), 758 (m), 743 (w), 722 (s), 701 (s), 652 (w), 621 (m), 608 (m), 555 (m), 532 (m), 509 (s), 438 (w).

**Elemental analysis**: Calcd. (%) C (41.85) H (7.17) N (12.95) F (27.02) P (11.01); Found (%) C (41.45) H (7.13) N (12.87) F (27.11) P (10.91).

**M.p.**: 180 °C (decomposition).


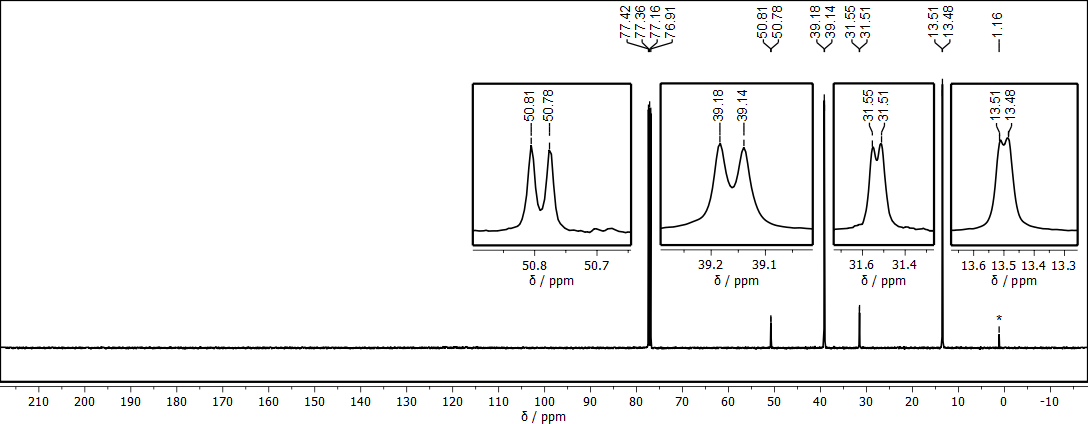


**Figure S 12.** ^13^C{^1^H} NMR spectrum of [EtP_4_H][P(C_2_F_5_)_2_(C_5_F_7_)F_3_] in CDCl_3_. Solvent signal 77.16 ppm.


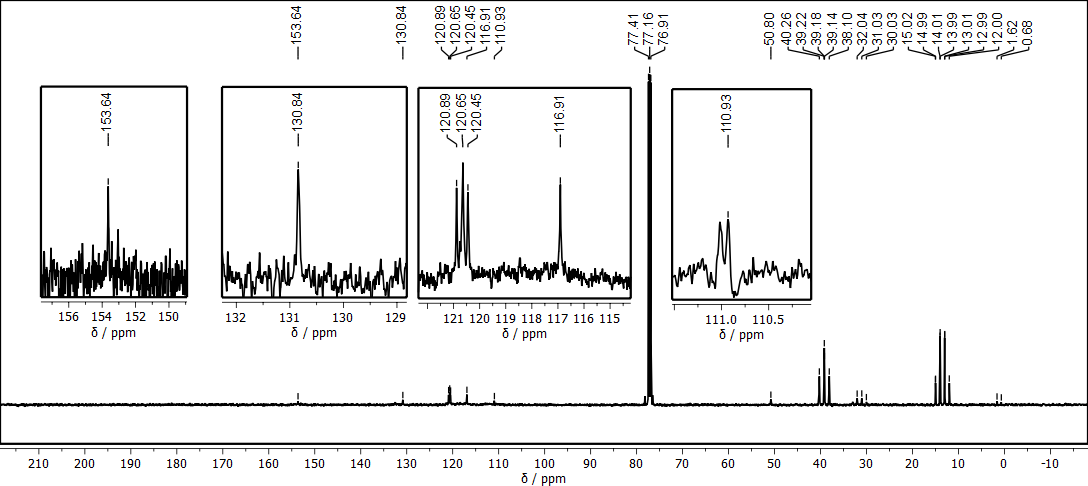


**Figure S 13.** ^13^C{^19^F} NMR spectrum of [EtP_4_H][P(C_2_F_5_)_2_(C_5_F_7_)F_3_] in CDCl_3_ (O1P = −80.0 ppm). Solvent signal 77.16 ppm.


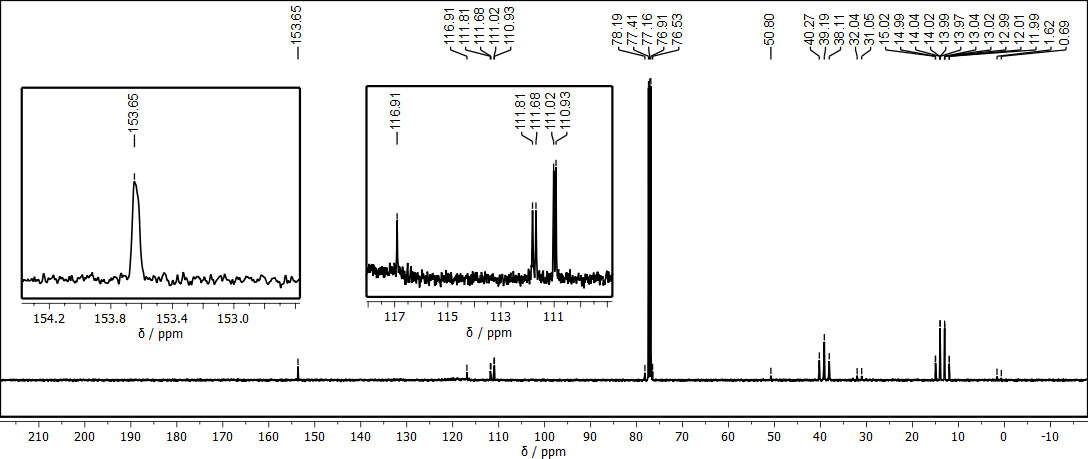


**Figure S 14.** ^13^C{^19^F} NMR spectrum of [EtP_4_H][P(C_2_F_5_)_2_(C_5_F_7_)F_3_] in CDCl_3_ (O1P = −130.0 ppm). Solvent signal 77.16 ppm.


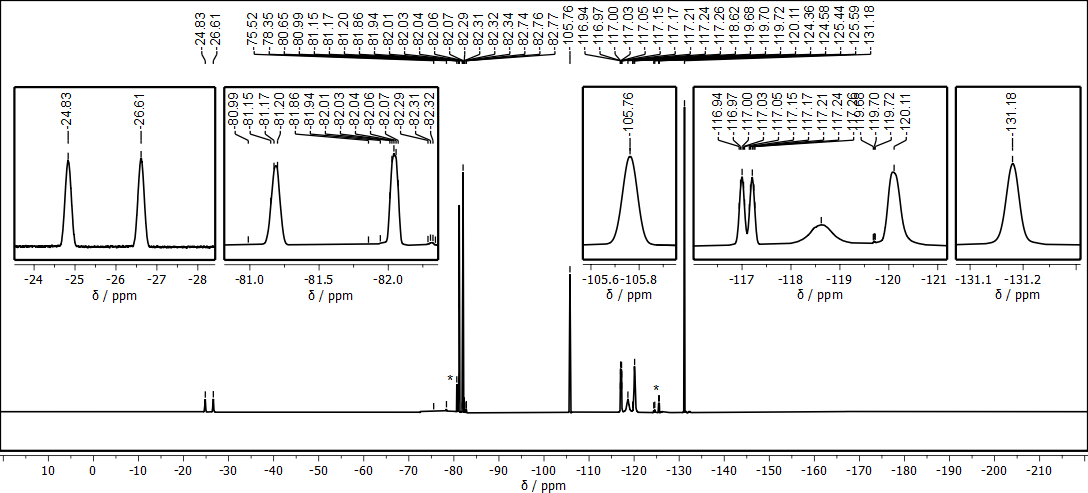


**Figure S 15.** ^19^F NMR spectrum of [EtP_4_H][P(C_2_F_5_)_2_(C_5_F_7_)F_3_] in CDCl_3_. *unknown impurity.


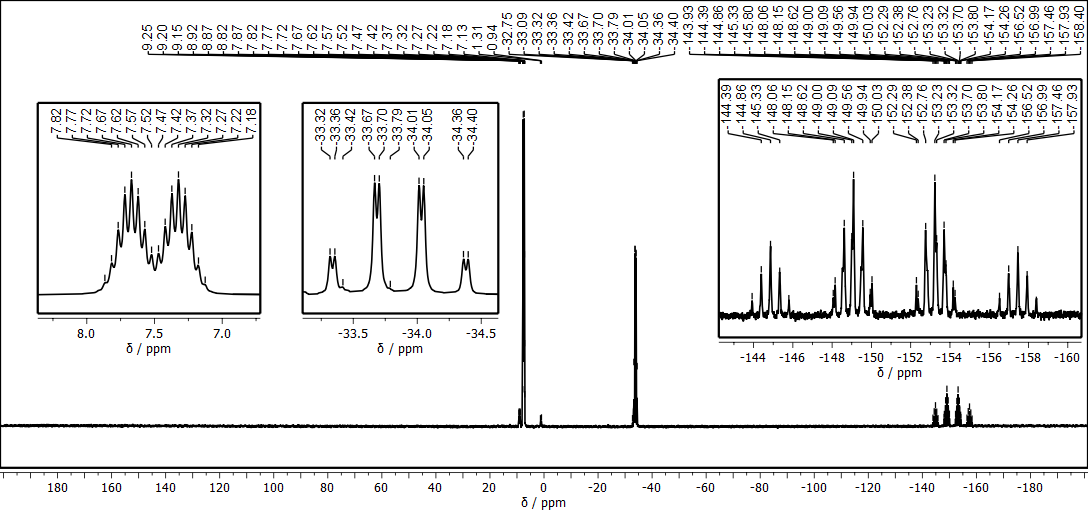


**Figure S 16.** ^31^P NMR spectrum of [EtP_4_H][P(C_2_F_5_)_2_(C_5_F_7_)F_3_] in CDCl_3_.

### **1.2.4** [EtP_4_H][P(C_2_F_5_)_2_(C_2_F_3_O)F_3_] (**2d**)

Onto a solution of [EtP_4_H][P(C_2_F_5_)_2_F_2_] (382 mg, 0.32 mmol) in Et_2_O (10 mL), C_2_F_6_O (0.75 mmol) was condensed. The reaction mixture was stirred for 17 h and all volatile components were removed. After recrystallization from a concentrated MeCN-solution at −30 °C, crystals were obtained and washed with *n*-pentane (2 x 8 mL). The product was obtained as a colorless solid (232 mg, 162 μmol, 50%). Crystals suitable for X-ray diffraction were obtained from a concentrated Et_2_O-solution at −30 °C.

**2d**: Cation omitted for clarity.

**^1^H NMR** (CDCl_3_, 303 K, 500 MHz): δ / ppm = 1.1 (t, ^3^*J*_H,H_ = 7 Hz, 54 H, CH_2_C**H_3_**), 1.3 (s, 9 H, C(C**H_3_**)_3_), 2.0 (d, ^2^*J*_H,P_ = 7 Hz, 1 H, N**H**), 3.1 (dquar, ^3^*J*_H,P_ = 10 Hz, ^3^*J*_H,H_ = 7 Hz, 36 H, C**H_2_**).

**^13^C{^1^H}** NMR (CDCl_3_, 303 K, 150 MHz): δ / ppm = 12.5 (d, ^3^*J*_C,P_ = 4 Hz, 6 C, CH_2_**C**H_3_), 30.5 (d, ^3^*J*_C,P_ = 5 Hz, 3 C, C(**C**H_3_)_3_), 38.2 (d, ^2^*J*_C,P_ = 5 Hz, 6 C, **C**H_2_), 49.8 (d, ^2^*J*_C,P_ = 4 Hz, 1 C, **C**(CH_3_)_3_).

**^13^C{^19^F}** NMR (CDCl_3_, 303 K, 150 MHz, O2P = −80 ppm): δ / ppm = 116.5 (d, ^2^*J*_C,P_ = 100 Hz, 1 C, C(O)**C**F_3_), 120.2 (d, ^2^*J*_C,P_ = 20 Hz, 2 C, **C**F_3_), 120.6 (d, ^2^*J*_C,P_ = 27 Hz, 2 C, **C**F_3_), 203.8 (d, ^1^*J*_C,P_ = 6 Hz, 1 C, **C**(O)).

No Signals caused by CF_2_-groups were observed.

**^19^F NMR** (CDCl_3_, 303 K, 470 MHz): δ / ppm = −117.6 (dm, ^2^*J*_F,P_ = 100 Hz, 2 F, C**F_2_**), −117.2 (dm, ^2^*J*_F,P_ = 91 Hz, 2 F, C**F_2_**), −89.2 (dm, ^1^*J*_F,P_ = 898 Hz, 2 F, P**F_2_**), −82.1 (m, 3 F, CF_2_C**F_3_**), −81.0 (m, 3 F, CF_2_C**F_3_**), −76.2 (m, 3 F, C(O)C**F_3_**), −47.0 (dm, ^1^*J*_F,P_ = 906 Hz, 1 F P**F**).

**^31^P NMR** (CDCl_3_, 303 K, 202 MHz): δ / ppm = −152.1 (dttt, ^1^*J*_F,P_ = 906 Hz, ^1^*J*_F,P_ = 898 Hz, ^2^*J*_F,P_ = 100 Hz, ^2^*J*_F,P_ = 91 Hz, 1 P, **P**F_3_), −33.8 (quartd, ^2^*J*_P,P_ = 70 Hz, ^2^*J*_H,P_ = 7 Hz, 1 P, **P**(NH*^t^*Bu)), 7.5 (dtridec, ^2^*J*_P,P_ = 70 Hz, ^2^*J*_H,P_ = 10 Hz, 3 P, **P**(NEt_2_)_3_).

**IR (ATR, solid)**: *ṽ* (cm^-1^): 2974 (w), 2937 (w), 2874 (w), 1736 (m), 1465 (w), 1379 (m), 1352 (w), 1276 (s), 1203 (s), 1172 (vs), 1143 (s), 1102 (m), 1056 (w), 1018 (vs), 941 (s), 849 (w), 812 (m), 793 (m), 611 (s), 753 (w), 703 (s), 619 (s), 577 (w), 508 (s), 481 (m), 448 (m).

**Elemental analysis**: Calcd. (%) C (42.17) H (7.69) N (13.90) O (1.22) F (23.20) P (11.82) Found (%) C (41.90) H (7.57) N (13.78) F (23.14) P (11.88) O (1.13).

**M.p.**: 142 °C-149 °C.


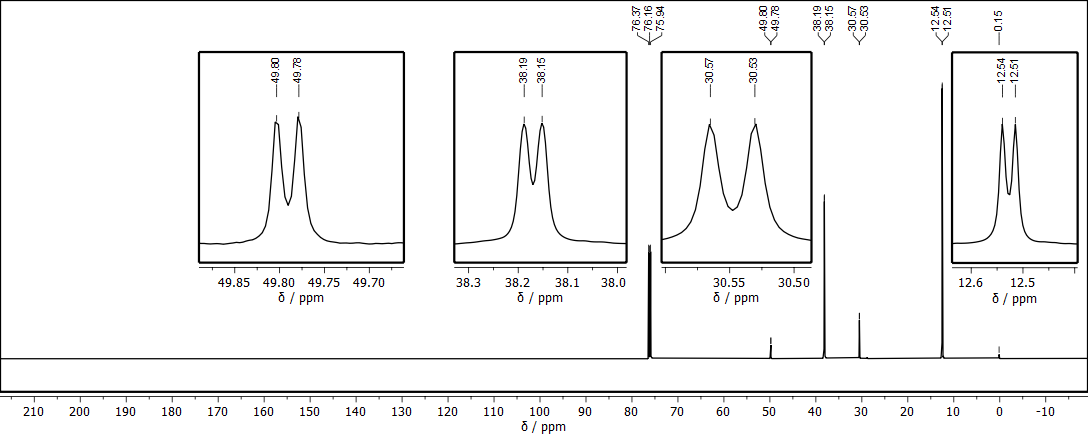


**Figure S 17.** ^13^C{^1^H} NMR spectrum of [EtP_4_H][P(C_2_F_5_)_2_(C_2_F_3_O)F_3_] in CDCl_3_. Solvent signal 77.16 ppm.


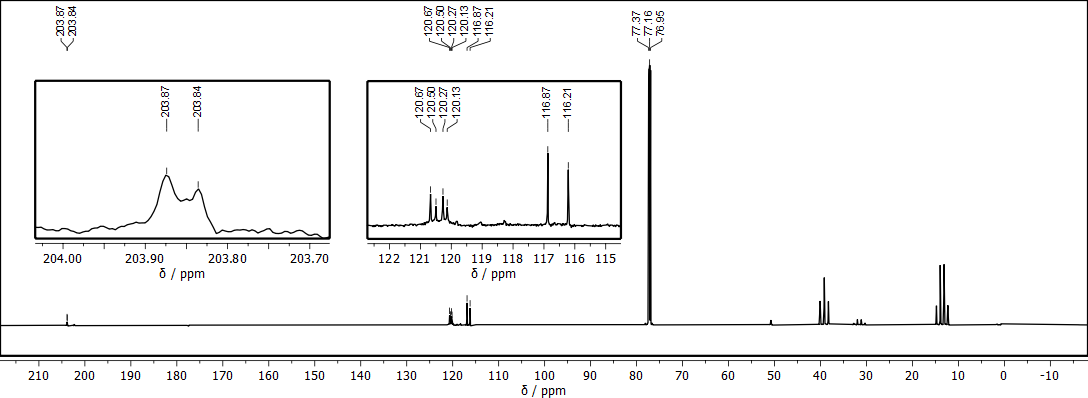


**Figure S 18.** ^13^C{^19^F} NMR spectrum of [EtP_4_H][P(C_2_F_5_)_2_(C_2_F_3_O)F_3_] in CDCl_3_ (O2P = −80.0 ppm). Solvent signal 77.16 ppm.


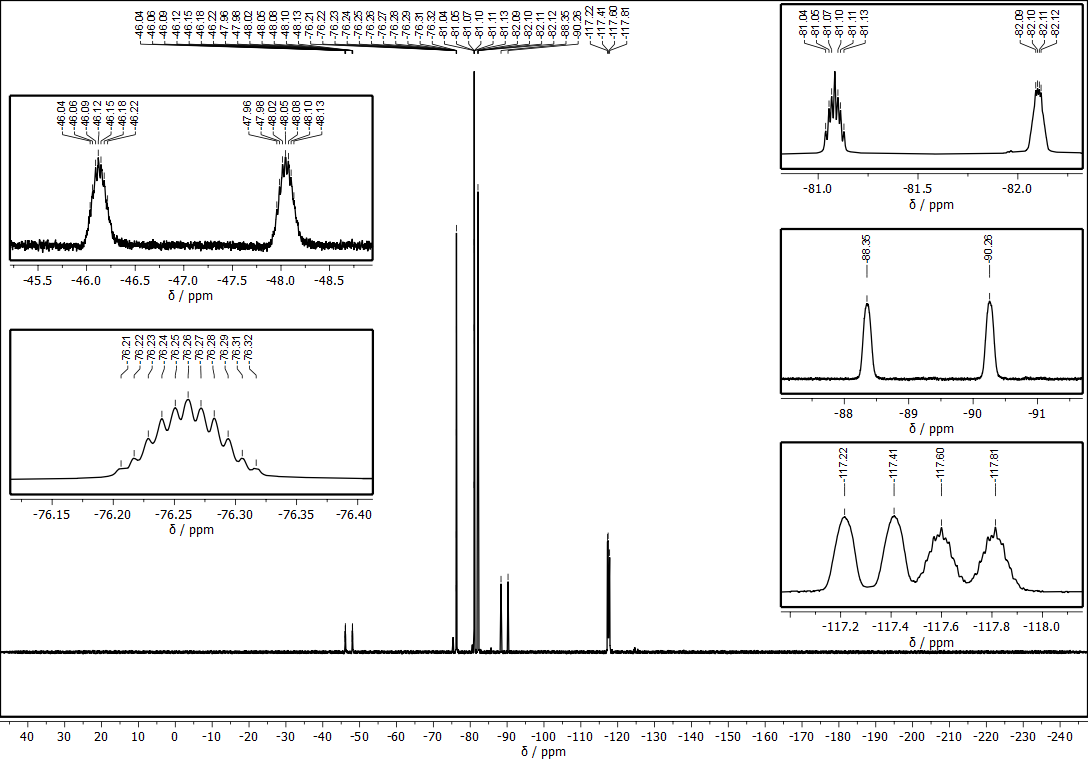


**Figure S 19.** ^19^F NMR spectrum of [EtP_4_H][P(C_2_F_5_)_2_(C_2_F_3_O)F_3_] in CDCl_3_.


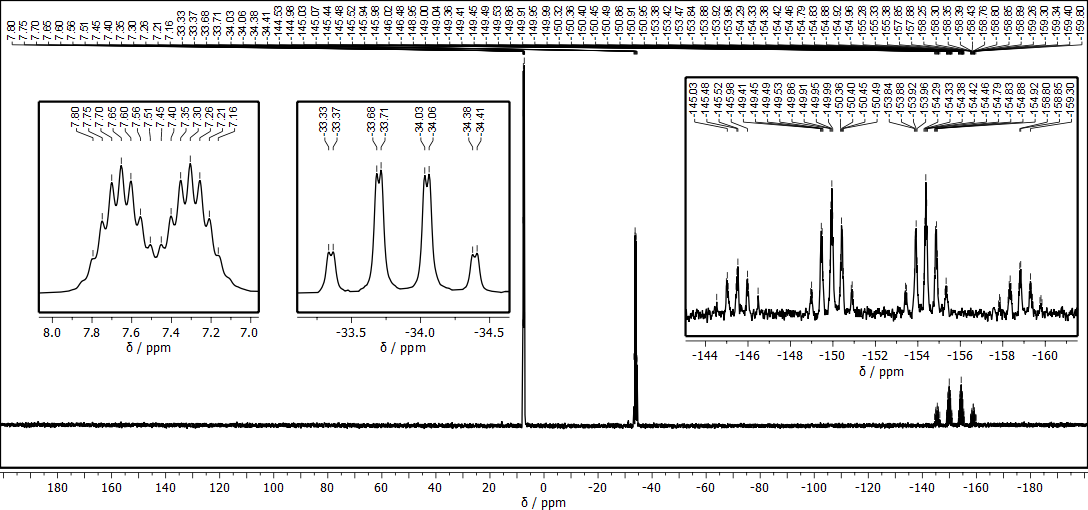


**Figure S 20.** ^31^P NMR spectrum of [EtP_4_H][P(C_2_F_5_)_2_(C_2_F_3_O)F_3_] in CDCl_3_.

### **1.2.5** [EtP_4_H][P(C_2_F_5_)_2_(C_3_F_5_O)F_3_] (**2e**)

Bromobis(pentafluoroethyl)phosphane (395 mg, 1.13 mmol) and MeCN (20 mL) were condensed on AgF (503 mg, 3.96 mmol) and stirred for 45 min after thawing. The black suspension was directly filtered on [EtP_4_H]Cl (873 mg, 0.95 mmol). The reaction mixture was stirred for 20 min and C_3_F_6_O (1.25 mmol) was condensed onto it. After 10 h the grey suspension was filtered and all volatile components were removed. Recrystallization from a concentrated MeCN-solution at −30 °C afforded crystals, which were washed with *n*-hexane (2 x 8 mL) and the product was obtained as a beige solid (395 mg, 0.29 mmol, 31%).

**2e**: Cation omitted for clarity.

**^1^H NMR** (CDCl_3_, 303 K, 500 MHz): δ / ppm = 1.0 (t, ^3^*J*_H,H_ = 7 Hz, 54 H, CH_2_C**H_3_**), 1.2 (s, 9 H, C(C**H_3_**)_3_), 1.9 (d, ^2^*J*_H,P_ = 7 Hz, 1 H, N**H**), 3.0 (dquar, ^3^*J*_H,P_ = 10 Hz, ^3^*J*_H,H_ = 7 Hz, 36 H, C**H_2_**).

**^13^C{^1^H} NMR** (CDCl_3_, 303 K, 150 MHz): δ / ppm = 12.5 (d, ^3^*J*_C,P_ = 4 Hz, 6 C, CH_2_**C**H_3_), 30.5 (d, ^3^*J*_C,P_ = 5 Hz, 3 C, C(**C**H_3_)_3_), 38.2 (d, ^2^*J*_C,P_ = 5 Hz, 6 C, **C**H_2_), 49.8 (d, ^2^*J*_C,P_ = 4 Hz, 1 C, **C**(CH_3_)_3_).

**^13^C{^19^F} NMR** (CDCl_3_, 303 K, 150 MHz): δ / ppm = 107.8 (d, ^2^*J*_C,P_ = 78 Hz, 1 C, C(O)**C**F_2_), 119.0 (d, ^3^*J*_C,P_ = 8 Hz, 1 C, C(O)CF_2_**C**F_3_), 120.2 (d, ^3^*J*_C,P_ = 21 Hz, 2 C, **C**F_3_), 120.6 (d, ^3^*J*_C,P_ = 27 Hz, 2 C, **C**F_3_), 205.7 (m, 1 C, **C**(O)).

Signals caused by CF_2_-groups located at the central phosphorus atom are overlapping with CF_3_ signals.

**^19^F NMR** (CDCl_3_, 303 K, 470 MHz): δ / ppm = −120.2 (m, 2 F, C(O)C**F_2_**), −117.7 (dm, ^2^*J*_F,P_ = 100 Hz, 2 F, C**F_2_**CF_3_), −117.0 (dm, ^2^*J*_F,P_ = 93 Hz, 2 F, C**F_2_**CF_3_), −88.7 (dm, ^1^*J*_F,P_ = 901 Hz, 2 F, P**F_2_**), −82.1 (m, 3 F, CF_2_C**F_3_**), −81.3 (m, 3 F, C(O)CF_2_C**F_3_**), −81.0 (m, 3 F, CF_2_C**F_3_**), −24.3 (dm, ^1^*J*_F,P_ = 910 Hz, 1 F, P**F**).

**^31^P NMR** (CDCl_3_, 303 K, 202 MHz): δ / ppm = −151.1 (tdtt, ^1^*J*_F,P_ = 908 Hz, ^1^*J*_F,P_ = 900 Hz, ^2^*J*_F,P_ = 100 Hz, ^2^*J*_F,P_ = 93 Hz, 1 P, **P**F_3_)), −33.8 (quartd, ^2^*J*_P,P_ = 70 Hz, ^2^*J*_H,P_ = 7 Hz, 1 P, **P**(NH*^t^*Bu)), 7.5 (dtridec, ^2^*J*_P,P_ = 70 Hz, ^2^*J*_H,P_ = 10 Hz, 3 P, **P**(NEt_2_)_3_).

**MS (ESI, pos., MeCN)**, *m/z* (%): 886 [EtP_4_H]^+^ (100).

**MS (ESI, neg., MeCN)**, *m/z* (%): 326 (28) [PF_3_(C_2_F_5_)_2_H]^−^, 473 (100) [PF_3_(C_2_F_5_)_2_(C_3_F_5_O)]^−^.

**IR (ATR, solid)**: *ṽ* (cm^-1^): 2974 (w), 2937 (w), 2874 (m), 1731 (w), 1466 (w), 1379 (m), 1351 (m), 1281 (s), 1204 (m), 1173 (vs), 1134 (m), 1103 (m), 1055 (w), 1018 (vs), 972 (s), 943 (s), 920 (w), 848 (w), 810 (m), 794 (m), 754 (w), 702 (s), 615 (s), 580 (w), 508 (s), 483 (m), 444 (m).

**Elemental analysis**: Calcd. (%) C (41.50) H (7.41) N (13.39) F (25.14) P (11.39) O (1.18); Found (%) C (41.39) H (7.44) N (13.38) F (25.17) P (11.37) O (1.14).

**M.p.**: 154 °C-160 °C (decomposition).


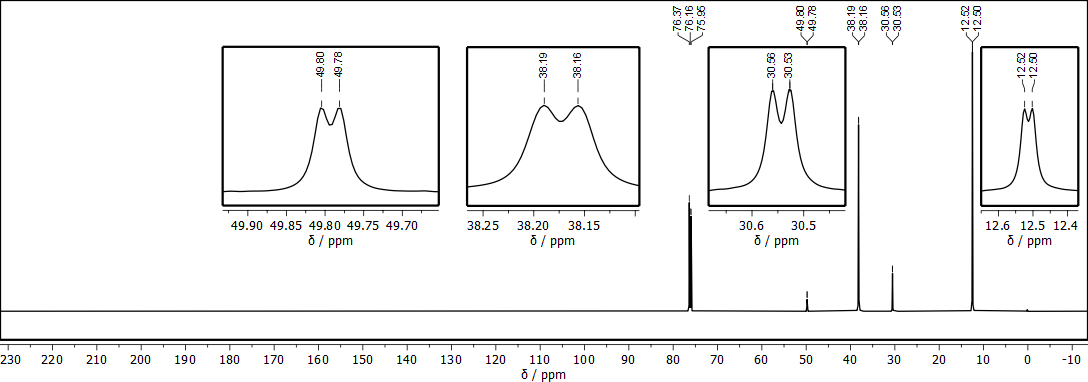


**Figure S 21.** ^13^C{^1^H} NMR spectrum of [EtP_4_H][P(C_2_F_5_)_2_(C_3_F_5_O)F_3_] in CDCl_3_. Solvent signal 77.16 ppm.


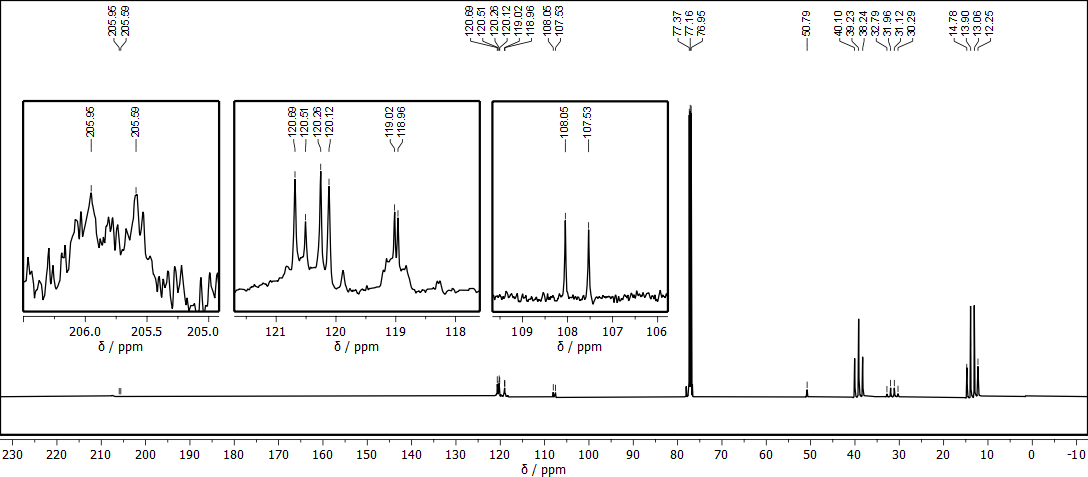


**Figure S 22.** ^13^C{^19^F} NMR spectrum of [EtP_4_H][P(C_2_F_5_)_2_(C_3_F_5_O)F_3_] in CDCl_3_ (O2P = −80.0 ppm). Solvent signal 77.16 ppm.


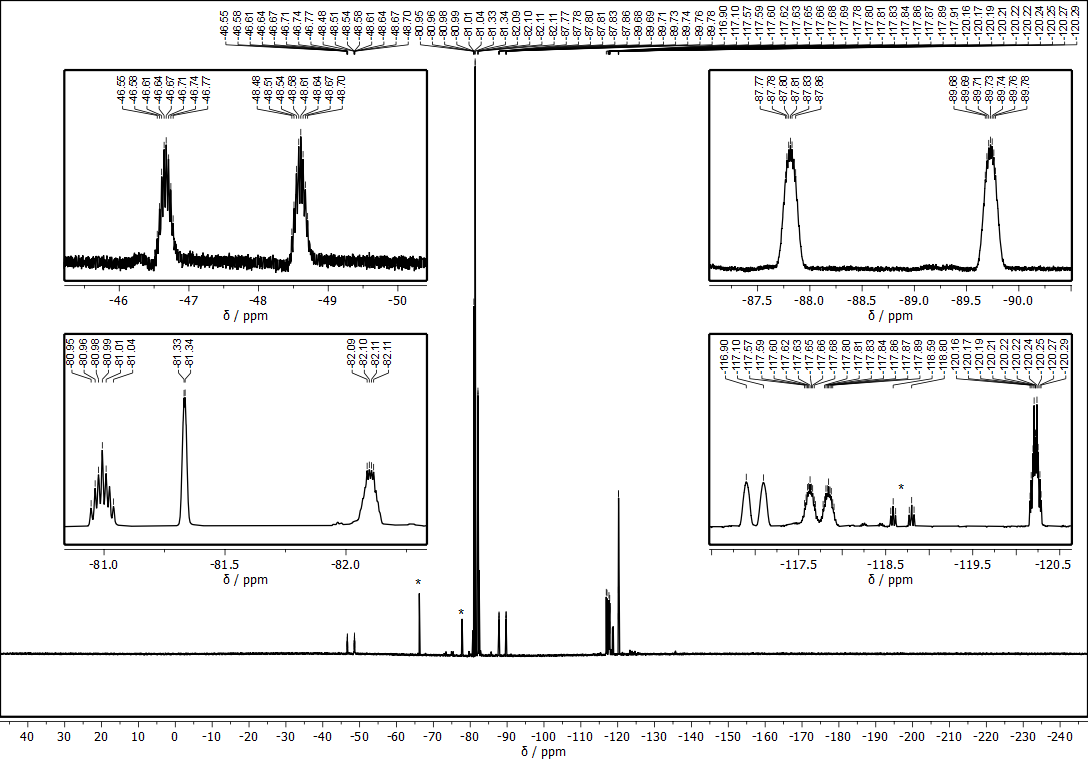


**Figure S 23.** ^19^F NMR spectrum of [EtP_4_H][P(C_2_F_5_)_2_(C_3_F_5_O)F_3_] in CDCl_3_. *unknown impurity.


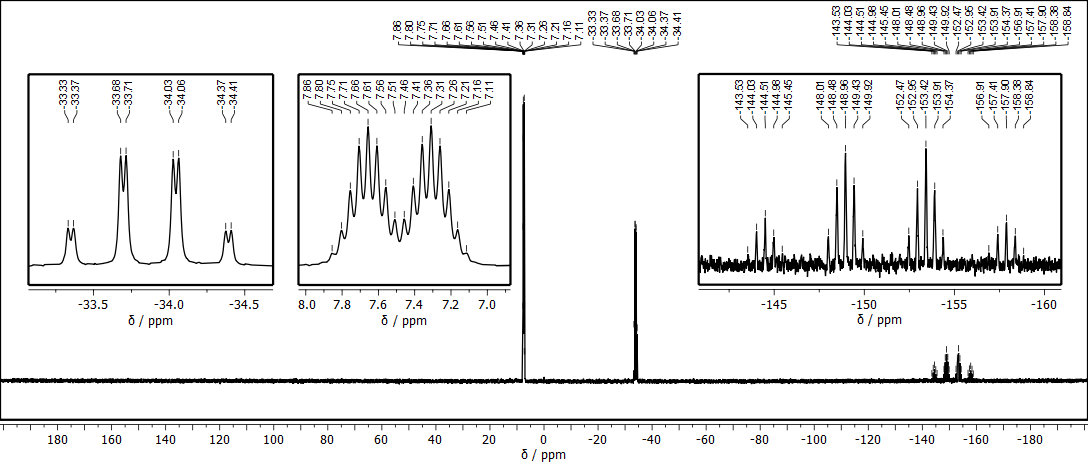


**Figure S 24.** ^31^P NMR spectrum of [EtP_4_H][P(C_2_F_5_)_2_(C_3_F_5_O)F_3_] in CDCl_3_.

# **2. X-ray Data**

Single crystal X-ray diffraction analyses were performed on a Rigaku Supernova diffractometer using Mo-K*α* or Cu-K*α* radiation. Crystals were kept at 100.0(1) K during data collection. Using Olex2, the structure was solved with SHELXT structure solution program using intrinsic phasing and refined with olex2.refine refinement package using Least square minimisation.^[36-38]^

Twinned crystal in phosphate **2a**, component 2 rotated by −179.9348% around [0.00 1.00 0.00] (reciprocal) or [−0.06 1.00 −0.02] (direct), BASF 0.1915(5). Disorder of C62, C63 over two sites in ratio 78:22. Disorder of fluorine atoms and C_2_F_5_ groups bonded to P5 over two sites in ratio 91:9. Suitable restraints and constraints were applied for the disordered atoms.

Disorder of nearly the entire cation and one CF_3_ group of phosphate **2b**. RIGU and SIMU restraints were used for disordered parts, SAME restraints for the P(NEt_2_)_3_ groups.

Twinned crystal in **2d**. Disorder of two ethyl groups of the cation over two sites in ratios of 55:45 and 82:18. The quality of the crystal was borderline.

CCDC 24993352-24993354 contain the supplementary crystallographic data for this paper. These data can be obtained free of charge from the Cambridge Crystallographic Data Centre via <https://www.ccdc.cam.ac.uk/structures>.

**Figure S 25.** Molecular structure in the solid state of phosphates **2b** and **2d**. A detailed discussion of structural parameters is not possible due to the use of many restraints and the depicted structures only act as a proof of connectivity.

**Table S1.** Selected bond lengths / pm of phosphate **2a**.

| **P-F^A^** | 161.1(2) and 161.7(2) |
| --- | --- |
| **P-F^B^** | 162.3(2) |
| **P-CF_2_^c^** | 195.2(3) |
| **P-CF_2_^d^** | 194.8(4) |
| **P-C^e^** | 192.6(3) |

**Table S2.** X-ray diffraction data of phosphates **2a,b** and **2d**.

|  | [EtP_4_H] [P(C_2_F_5_)_2_(C_5_NF_4_)F_3_]  **2a** | [EtP_4_H] [P(C_2_F_5_)_2_(C_3_F_5_)F_3_]  **2b** | [EtP_4_H] [P(C_2_F_5_)_2_(C_2_F_3_O)F_3_]  **2d** |
| --- | --- | --- | --- |
| Empirical Formula | C_49_H_100_F_17_N_14_P_5_ | C_47_H_100_F_18_N_13_P_5_ | C_47_H_99_F_18_N_13_OP_5_ |
| Formula weight/g∙mol^-1^ | 1363.27 | 1344.24 | 1359.24 |
| Temperature/K | 100.0(1) | 100.0(1) | 100.0(1) |
| Crystal system | triclinic | triclinic | triclinic |
| Space group | *P*-1 | *P*-1 | *P*-1 |
| a/Å | 13.0277(2) | 13.2508(3) | 13.1511(6) |
| b/Å | 14.7296(2) | 14.3862(3) | 14.5039(9) |
| c/Å | 34.5182(4) | 17.1831(3) | 17.4474(7) |
| *α*/° | 86.6398(13) | 87.093(1) | 84.574(4) |
| *β*/° | 85.3139(14) | 86.818(2) | 87.730(4) |
| *γ*/° | 86.6755(14) | 88.290(2) | 88.303(4) |
| Volume/Å^3^ | 6580.89(19) | 3265.1(1) | 3309.3(3) |
| Z | 4 | 2 | 2 |
| *ρ*_calc_g/cm^3^ | 1.376 | 1.367 | 1.364 |
| *μ*/mm^‑1^ | 0.233 | 2.138 | 2.128 |
| F(000) | 2880.0 | 1420.0 | 1434.0 |
| Crystal size/mm^3^ | 0.619 × 0.197 × 0.15 | 0.3 × 0.28 × 0.1 | 0.29 × 0.224 × 0.191 |
| Radiation/Å | Mo K*α* (*λ* = 0.71073) | Cu K*α* (*λ* = 1.54184) | Cu K*α* (*λ* = 1.54184) |
| 2Θ range for data collection/° | 6.23444 to 60.162 | 5.158 to 153.992 | 6.124 to 151.766 |
| Index ranges | -18 ≤ h ≤ 18, -20 ≤ k ≤ 20, -48 ≤ l ≤ 48 | -16 ≤ h ≤ 16, -18 ≤ k ≤ 18, -21 ≤ l ≤ 21 | -14 ≤ h ≤ 16, -17 ≤ k ≤ 18, -20 ≤ l ≤ 21 |
| Reflections collected | 196015 | 93343 | 27569 |
| Independent reflections | 62940 [R_int_ = 0.0562, R_sigma_ = 0.1037] | 13593 [R_int_ = 0.0520, R_sigma_ = 0.0237] | 13333 [R_int_ = 0.0487, R_sigma_ = 0.0576] |
| Reflections with *I>=2u(I)* | 45476 | 11740 | 9243 |
| Data/restraints/ parameters | 62940/420/1745 | 13593/2340/1245 | 13333/897/949 |
| Goodness-of-fit on F^2^ | 1.054 | 1.753 | 1.564 |
| Final R indexes [ *I>=2u(I)*] | R_1_ = 0.0700, wR_2_ = 0.2027 | R_1_ = 0.1069, wR_2_ = 0.3562 | R_1_ = 0.1220, wR_2_ = 0.3614 |
| Final R indexes [all data] | R_1_ = 0.0945, wR_2_ = 0.2168 | R_1_ = 0.1145, wR_2_ = 0.3747 | R_1_ = 0.1534, wR_2_ = 0.4073 |
| Largest diff. peak/hole / e Å^-3^ | 0.82/-0.63 | 1.34/-0.58 | 1.46/-0.89 |
| CCDC | 2499352 | 2499353 | 2499354 |

# **3. References**

[36] L. J. Bourhis, O. V. Dolomanov, R. J. Gildea, J. A. K. Howard, H. Puschmann, *Acta Crystallogr, Sect. A: Found. Adv.* **2015**, *71*, 59–75.

[37] G. M. Sheldrick, *Acta Crystallogr, Sect. C: Struct. Chem.* **2015**, *71*, 3–8.

[38] O. V. Dolomanov, L. J. Bourhis, R. J. Gildea, J. A. K. Howard, H. Puschmann, *J. Appl. Crystallogr.* **2009**, *42*, 339–341.
