# Supplementary material for: Oxidative Addition of C‐F Bonds to the Phosphoranide Ion [P(C2F5)2F2]−
Source: Chemistry. 2025 Dec 12;32(2):e03405. doi: 10.1002/chem.202503405 (PMC12790317; doi:10.1002/chem.202503405)

checkCIF/PLATON report

Structure factors have been supplied for datablock(s) 2a

THIS REPORT IS FOR GUIDANCE ONLY. IF USED AS PART OF A REVIEW PROCEDURE FOR PUBLICATION, IT SHOULD NOT REPLACE THE EXPERTISE OF AN EXPERIENCED CRYSTALLOGRAPHIC REFEREE.

No syntax errors found. CIF dictionary Interpreting this report

Datablock: 2a

```
Bond precision:   C-C = 0.0050 A          Wavelength=0.71073

Cell:            a=13.0277(2)        b=14.7296(2)       c=34.5182(5)
                 alpha=86.6398(13)   beta=85.3139(14)   gamma=86.6755(14)
Temperature:     100 K

                   Calculated                   Reported
Volume             6580.88(17)                  6580.89(19)
Space group        P -1                         P -1
Hall group         -P 1                         -P 1
Moiety formula     C40 H100 N13 P4, C9 F17 N P  C40 H100 N13 P4, C9 F17 N P
Sum formula        C49 H100 F17 N14 P5          C49 H100 F17 N14 P5
Mr                 1363.28                      1363.27
Dx,g cm-3          1.376                        1.376
Z                  4                            4
Mu (mm-1)          0.233                        0.233
F000               2880.0                       2880.0
F000'              2883.66
h,k,lmax           18,20,48                     18,20,48
Nref               38667                        62940
Tmin,Tmax          0.946,0.966                  0.302,1.000
Tmin'              0.866

Correction method= # Reported T Limits: Tmin=0.302 Tmax=1.000
AbsCorr = GAUSSIAN
```

```
Data completeness= 1.628            Theta(max)= 30.081

R(reflections)= 0.0700( 45476)               wR2(reflections)=
                                             0.2168( 62940)
S = 1.054                   Npar= 1745
```

The following ALERTS were generated. Each ALERT has the format
test-name_ALERT_alert-type_alert-level.
Click on the hyperlinks for more details of the test.

Alert level B

```
PLAT910_ALERT_3_B Missing FCF Reflection(s) Below Theta(Min)[Deg]=       3.11 Note
                1  0  0,  -1  1  0,   0  1  0,   1  1  0,   0  2  0,   0 -2  1,
               -1 -1  1,   0 -1  1,   1 -1  1,  -1  0  1,   0  0  1,   1  0  1,
               -1  1  1,   0  1  1,   1  1  1,   0  2  1,   0 -2  2,  -1 -1  2,
                0 -1  2,   1 -1  2,  -1  0  2,   0  0  2,   1  0  2,  -1  1  2,
                  (   19 More Missing: see the .ckf listing file)
```

Author Response: Can be expected if a beam catcher is used.

Alert level C

```
PLAT220_ALERT_2_C NonSolvent   Resd 1  C   Ueq(max)/Ueq(min) Range        3.5 Ratio
PLAT230_ALERT_2_C Hirshfeld Test Diff for    P1       --N1       .        5.5 s.u.
PLAT340_ALERT_3_C Low Bond Precision on  C-C Bonds ...............    0.00496 Ang.
PLAT420_ALERT_2_C D-H Bond Without Acceptor  N1       --H1       .     Please Check
PLAT420_ALERT_2_C D-H Bond Without Acceptor  N15      --H15      .     Please Check
PLAT906_ALERT_3_C Large K Value in the Analysis of Variance ......      3.165 Check
PLAT911_ALERT_3_C Missing FCF Refl Between Thmin & STh/L=    0.600         17 Report
                2  0  0,  -1  2  0,   0  7  0,   2  0  1,  -1 12  1,   0 -5  2,
                2  0  2,  -2-12  3,   0  1  5,   0  0 13,  11 -6 15,   0 -2 16,
                0  1 17,  -9  0 24,   7 -1 26,  -4  2 29,  -3  3 30,
PLAT918_ALERT_3_C Reflection(s) with I(obs) much Smaller I(calc) .          2 Check
```

Alert level G

```
PLAT002_ALERT_2_G Number of Distance or Angle Restraints on AtSite         32 Note
PLAT003_ALERT_2_G Number of Uiso or U(i,j)  Restrained non-H-Atoms         34 Report
PLAT063_ALERT_4_G Crystal Size Possibly too Large for Beam Size ..       0.62 mm
PLAT072_ALERT_2_G SHELXL First  Parameter in WGHT  Unusually Large       0.13 Report
PLAT152_ALERT_1_G The Supplied and Calc. Volume s.u. Differ by ...         -2 Units
PLAT176_ALERT_4_G The CIF-Embedded .res File Contains SADI Records         36 Report
PLAT178_ALERT_4_G The CIF-Embedded .res File Contains SIMU Records          5 Report
PLAT187_ALERT_4_G The CIF-Embedded .res File Contains RIGU Records          4 Report
PLAT188_ALERT_3_G A Non-default SIMU Restraint Value has been used     0.0010 Report
PLAT188_ALERT_3_G A Non-default SIMU Restraint Value has been used     0.0010 Report
PLAT188_ALERT_3_G A Non-default SIMU Restraint Value has been used     0.0010 Report
PLAT188_ALERT_3_G A Non-default SIMU Restraint Value has been used     0.0010 Report
PLAT188_ALERT_3_G A Non-default SIMU Restraint Value has been used     0.0010 Report
PLAT190_ALERT_3_G A Non-default RIGU Restraint Value for First Par     0.0010 Report
PLAT190_ALERT_3_G A Non-default RIGU Restraint Value for SecondPar     0.0010 Report
```

```
PLAT190_ALERT_3_G A Non-default RIGU Restraint Value for First Par     0.0010 Report
PLAT190_ALERT_3_G A Non-default RIGU Restraint Value for SecondPar     0.0010 Report
PLAT190_ALERT_3_G A Non-default RIGU Restraint Value for First Par     0.0010 Report
PLAT190_ALERT_3_G A Non-default RIGU Restraint Value for SecondPar     0.0010 Report
PLAT190_ALERT_3_G A Non-default RIGU Restraint Value for First Par     0.0010 Report
PLAT190_ALERT_3_G A Non-default RIGU Restraint Value for SecondPar     0.0010 Report
PLAT191_ALERT_3_G A Non-default SADI Restraint Value has been used     0.0400 Report
PLAT191_ALERT_3_G A Non-default SADI Restraint Value has been used     0.0400 Report
PLAT191_ALERT_3_G A Non-default SADI Restraint Value has been used     0.0400 Report
PLAT191_ALERT_3_G A Non-default SADI Restraint Value has been used     0.0400 Report
PLAT191_ALERT_3_G A Non-default SADI Restraint Value has been used     0.0400 Report
PLAT191_ALERT_3_G A Non-default SADI Restraint Value has been used     0.0400 Report
PLAT191_ALERT_3_G A Non-default SADI Restraint Value has been used     0.0400 Report
PLAT191_ALERT_3_G A Non-default SADI Restraint Value has been used     0.0400 Report
PLAT191_ALERT_3_G A Non-default SADI Restraint Value has been used     0.0400 Report
PLAT191_ALERT_3_G A Non-default SADI Restraint Value has been used     0.0400 Report
PLAT191_ALERT_3_G A Non-default SADI Restraint Value has been used     0.0400 Report
PLAT191_ALERT_3_G A Non-default SADI Restraint Value has been used     0.0400 Report
PLAT191_ALERT_3_G A Non-default SADI Restraint Value has been used     0.0400 Report
PLAT191_ALERT_3_G A Non-default SADI Restraint Value has been used     0.0400 Report
PLAT191_ALERT_3_G A Non-default SADI Restraint Value has been used     0.0400 Report
PLAT191_ALERT_3_G A Non-default SADI Restraint Value has been used     0.0400 Report
PLAT191_ALERT_3_G A Non-default SADI Restraint Value has been used     0.0400 Report
PLAT191_ALERT_3_G A Non-default SADI Restraint Value has been used     0.0400 Report
PLAT191_ALERT_3_G A Non-default SADI Restraint Value has been used     0.0400 Report
PLAT191_ALERT_3_G A Non-default SADI Restraint Value has been used     0.0400 Report
PLAT191_ALERT_3_G A Non-default SADI Restraint Value has been used     0.0400 Report
PLAT191_ALERT_3_G A Non-default SADI Restraint Value has been used     0.0400 Report
PLAT191_ALERT_3_G A Non-default SADI Restraint Value has been used     0.0400 Report
PLAT230_ALERT_2_G Hirshfeld Test Diff for    F21      --C90      .        5.2 s.u.
PLAT230_ALERT_2_G Hirshfeld Test Diff for    F26      --C92      .        6.0 s.u.
PLAT242_ALERT_2_G Low    'MainMol' Ueq as Compared to Neighbors of        C42 Check
PLAT301_ALERT_3_G Main Residue  Disorder .............(Resd    1)          4% Note
PLAT302_ALERT_4_G Anion/Solvent/Minor-Residue Disorder (Resd    3)        57% Note
PLAT412_ALERT_2_G Short Intra XH3 .. XHn     H61C     ..H63F     .       1.96 Ang.
                                                  x,y,z  =      1_555 Check
PLAT802_ALERT_4_G CIF Input Record(s) with more than 80 Characters          2 Info
PLAT860_ALERT_3_G Number of Least-Squares Restraints ............         420 Note
PLAT870_ALERT_4_G ALERTS Related to Twinning Effects Suppressed ..          ! Info
PLAT912_ALERT_4_G Missing # of FCF Reflections Above STh/L=  0.600          2 Note
PLAT913_ALERT_3_G Missing # of Very Strong Reflections in FCF ....          1 Note
                0  0  4,
PLAT931_ALERT_5_G CIFcalcFCF  Twin Law ( 0 1 0)         Est.d BASF       0.21 Check
PLAT933_ALERT_2_G Number of HKL-OMIT Records in Embedded .res File         25 Note
               -7  1-26,  -6 -2 -4,  -6  2 -2,  -4  2 29,  -3-20-15,  -1 -1-28,
               -1  0 13,  -1  8  5,  -1 12  1,   0 -7  0,   0 -3  2,   0 -2 16,
                0 -1-17,   0 -1-15,   0  0-13,   0  0 13,   0  5 -2,   1  0 29,
                1  5 42,   2  1 -3,   2 12 -3,   3 -3-30,   8  7 25,   9  0-24,
               11 -6 15,
PLAT941_ALERT_3_G Average HKL Measurement Multiplicity ..........        1.6 Low
PLAT969_ALERT_5_G The 'Henn et al.' R-Factor-gap value ..........      8.881 Note
              Predicted wR2: Based on SigI**2  2.44 or SHELX Weight 20.57
```

---

0 **ALERT level A** = Most likely a serious problem - resolve or explain
1 **ALERT level B** = A potentially serious problem, consider carefully
8 **ALERT level C** = Check. Ensure it is not caused by an omission or oversight

```
59 ALERT level G = General information/check it is not something unexpected

 1 ALERT type 1 CIF construction/syntax error, inconsistent or missing data
12 ALERT type 2 Indicator that the structure model may be wrong or deficient
45 ALERT type 3 Indicator that the structure quality may be low
 8 ALERT type 4 Improvement, methodology, query or suggestion
 2 ALERT type 5 Informative message, check
```

It is advisable to attempt to resolve as many as possible of the alerts in all categories. Often the minor alerts point to easily fixed oversights, errors and omissions in your CIF or refinement strategy, so attention to these fine details can be worthwhile. It is up to the individual to critically assess their own results and, if necessary, seek expert advice.

PLATON version of 04/06/2025; check.def file version of 30/05/2025

---

duplicate check

No duplication found

Datablock 2a - ellipsoid plot

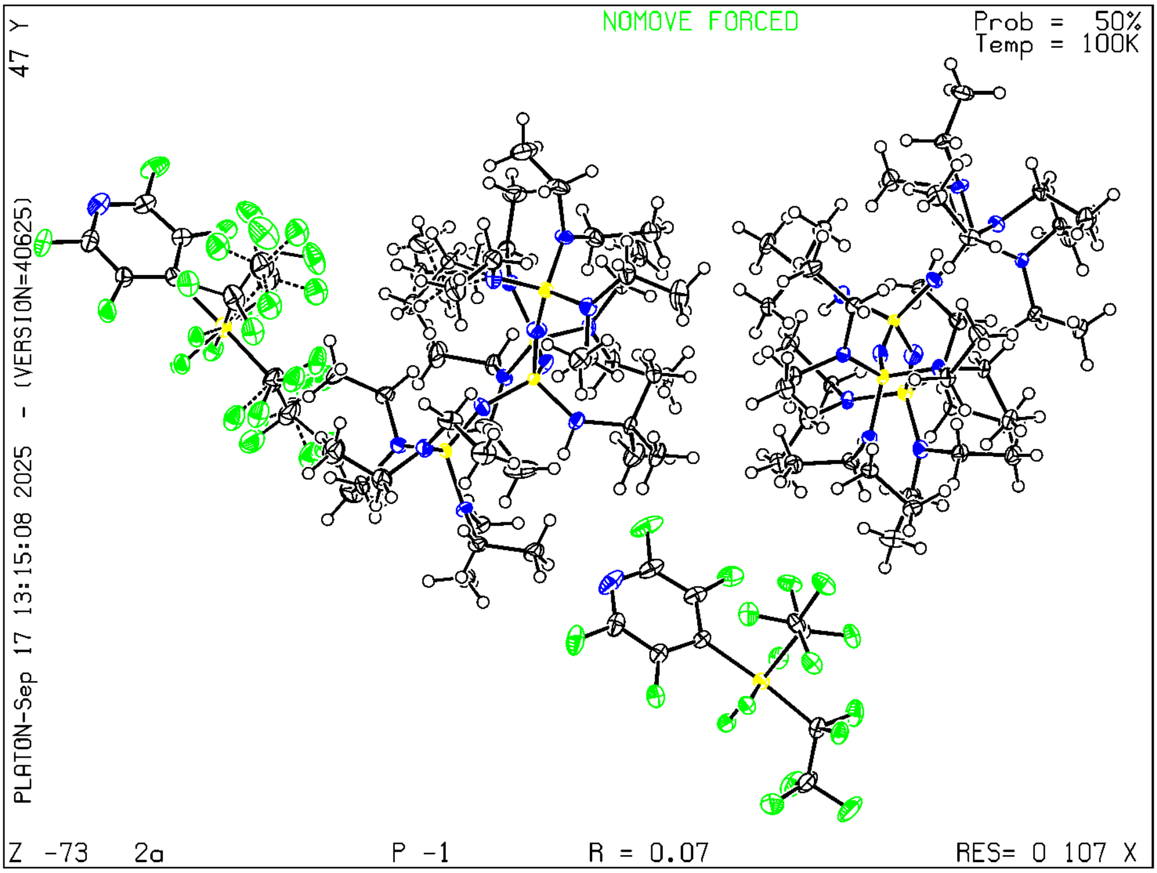

Supplement: Supplementary file 2 — Supporting Information file 2: chem70542‐sup‐0002‐DataFile.zip [file CHEM-32-e03405-s001.zip › checkcif_2a.pdf]
