# Supplementary material for: Oxidative Addition of C‐F Bonds to the Phosphoranide Ion [P(C2F5)2F2]−
Source: Chemistry. 2025 Dec 12;32(2):e03405. doi: 10.1002/chem.202503405 (PMC12790317; doi:10.1002/chem.202503405)

checkCIF/PLATON report

Structure factors have been supplied for datablock(s) 2b

THIS REPORT IS FOR GUIDANCE ONLY. IF USED AS PART OF A REVIEW PROCEDURE FOR PUBLICATION, IT SHOULD NOT REPLACE THE EXPERTISE OF AN EXPERIENCED CRYSTALLOGRAPHIC REFEREE.

No syntax errors found. CIF dictionary Interpreting this report

Datablock: 2b

```
Bond precision:   C-C = 0.0074 A          Wavelength=1.54184

Cell:             a=13.2508(3)      b=14.3862(3)      c=17.1831(3)
                  alpha=87.093(1)   beta=86.818(2)    gamma=88.290(2)
Temperature:      100 K

                   Calculated                    Reported
Volume             3265.13(12)                   3265.13(12)
Space group        P -1                          P -1
Hall group         -P 1                          -P 1
Moiety formula     C40 H100 N13 P4, C7 F18 P     C7 F18 P, C40 H100 N13 P4
Sum formula        C47 H100 F18 N13 P5           C47 H100 F18 N13 P5
Mr                 1344.25                       1344.24
Dx,g cm-3          1.367                         1.367
Z                  2                             2
Mu (mm-1)          2.138                         2.138
F000               1420.0                        1420.0
F000'              1427.84
h,k,lmax           16,18,21                      16,18,21
Nref               13805                         13593
Tmin,Tmax          0.553,0.808                   0.519,1.000
Tmin'              0.501

Correction method= # Reported T Limits: Tmin=0.519 Tmax=1.000
AbsCorr = GAUSSIAN
```

Data completeness= 0.985 Theta(max)= 76.996

R(reflections)= 0.1069(11740) wR2(reflections)= 0.3747(13593)

S = 1.753 Npar= 1245

The following ALERTS were generated. Each ALERT has the format
test-name_ALERT_alert-type_alert-level.
Click on the hyperlinks for more details of the test.

Alert level B

```
PLAT084_ALERT_3_B High wR2 Value (i.e. > 0.25) ..................       0.37 Report
```

Author Response: Due to low quality of the crystal and the heavy disorder of nearly the entire cation.

Alert level C

```
DIFMX02_ALERT_1_C  The maximum difference density is > 0.1*ZMAX*0.75
            The relevant atom site should be identified.
PLAT042_ALERT_1_C Calc. and Reported MoietyFormula Strings  Differ     Please Check
              Calc: C40 H100 N13 P4, C7 F18 P
              Rep.: C7 F18 P, C40 H100 N13 P4
PLAT082_ALERT_2_C High R1 Value .................................       0.11 Report
PLAT094_ALERT_2_C Ratio of Maximum / Minimum Residual Density ....       2.31 Report
PLAT097_ALERT_2_C Large Reported Max.  (Positive) Residual Density       1.34 eA-3
PLAT220_ALERT_2_C NonSolvent   Resd 1  C    Ueq(max)/Ueq(min) Range       4.2 Ratio
PLAT222_ALERT_3_C NonSolvent Resd 1  H    Uiso(max)/Uiso(min) Range       5.3 Ratio
PLAT242_ALERT_2_C Low    'MainMol' Ueq as Compared to Neighbors of        C4B Check
PLAT340_ALERT_3_C Low Bond Precision on  C-C Bonds ...............    0.00743 Ang.
PLAT420_ALERT_2_C D-H Bond Without Acceptor  N3B      --H3B      .     Please Check
PLAT906_ALERT_3_C Large K Value in the Analysis of Variance ......      3.122 Check
PLAT911_ALERT_3_C Missing FCF Refl Between Thmin & STh/L=    0.600         17 Report
                0   2   0,  -1 -2   1,  -1 -1   1,   1 -1   1,  -1  1   1,   1  1   1,
               -1   2   1,   0  0   2,  -2 -2   3,   0  0   4,  -2 -2   5,  -1  5   5,
               -2   1   6, -13 -3  10, -12 -3  11, -12 -3  12, -12 -2  12,
PLAT913_ALERT_3_C Missing # of Very Strong Reflections in FCF ....          5 Note
                0   2   0,  -1 -1   1,  -1  1   1,  -1  2   1,   0  0   2,
PLAT918_ALERT_3_C Reflection(s) with I(obs) much Smaller I(calc) .          2 Check
```

Alert level G

```
PLAT002_ALERT_2_G Number of Distance or Angle Restraints on AtSite         80 Note
PLAT003_ALERT_2_G Number of Uiso or U(i,j)  Restrained non-H-Atoms        110 Report
PLAT007_ALERT_5_G Number of Unrefined Donor-H Atoms ..............          2 Report
              H3B   H3A
PLAT072_ALERT_2_G SHELXL First  Parameter in WGHT  Unusually Large       0.20 Report
PLAT175_ALERT_4_G The CIF-Embedded .res File Contains SAME Records          4 Report
PLAT178_ALERT_4_G The CIF-Embedded .res File Contains SIMU Records          1 Report
PLAT187_ALERT_4_G The CIF-Embedded .res File Contains RIGU Records          1 Report
```

```
PLAT230_ALERT_2_G Hirshfeld Test Diff for    P11B     --N8B      .        7.7 s.u.
PLAT230_ALERT_2_G Hirshfeld Test Diff for    P27B     --N33B     .        5.5 s.u.
PLAT230_ALERT_2_G Hirshfeld Test Diff for    P43B     --N44B     .       10.2 s.u.
PLAT242_ALERT_2_G Low    'MainMol' Ueq as Compared to Neighbors of         C3 Check
PLAT242_ALERT_2_G Low    'MainMol' Ueq as Compared to Neighbors of         C5 Check
PLAT242_ALERT_2_G Low    'MainMol' Ueq as Compared to Neighbors of         C7 Check
PLAT301_ALERT_3_G Main Residue  Disorder .............(Resd    1)        91% Note
PLAT302_ALERT_4_G Anion/Solvent/Minor-Residue Disorder (Resd    2)        12% Note
PLAT412_ALERT_2_G Short Intra XH3 .. XHn     H5BB     ..H20B     .       1.99 Ang.
                                                      x,y,z  =      1_555 Check
PLAT412_ALERT_2_G Short Intra XH3 .. XHn     H6BB     ..H45A     .       2.04 Ang.
                                                      x,y,z  =      1_555 Check
PLAT412_ALERT_2_G Short Intra XH3 .. XHn     H6BC     ..H13D     .       2.09 Ang.
                                                      x,y,z  =      1_555 Check
PLAT413_ALERT_2_G Short Inter XH3 .. XHn     H5BA     ..H32D     .       2.08 Ang.
                                                 1-x,-y,1-z  =      2_656 Check
PLAT720_ALERT_4_G Number of Unusual/Non-Standard Labels ..........          9 Note
              H5BA    H5BB    H5BC    H6BA    H6BB    H6BC    H7BA    H7BB
              H7BC
PLAT790_ALERT_4_G Centre of Gravity not Within Unit Cell: Resd.  #          2 Note
              C7 F18 P
PLAT802_ALERT_4_G CIF Input Record(s) with more than 80 Characters          1 Info
PLAT811_ALERT_5_G No ADDSYM Analysis: Too Many Excluded Atoms ....          ! Info
PLAT860_ALERT_3_G Number of Least-Squares Restraints ............        2340 Note
PLAT912_ALERT_4_G Missing # of FCF Reflections Above STh/L=  0.600        195 Note
PLAT933_ALERT_2_G Number of HKL-OMIT Records in Embedded .res File          5 Note
               -1  5  5,  -2 -2  5,  -2 -2  3,   0  0  4,  -2  1  6,
PLAT969_ALERT_5_G The 'Henn et al.' R-Factor-gap value ..........      12.013 Note
              Predicted wR2: Based on SigI**2  3.12 or SHELX Weight 21.37
PLAT978_ALERT_2_G Number C-C Bonds with Positive Residual Density.          0 Info
PLAT992_ALERT_5_G Repd & Actual _reflns_number_gt Values Differ by          2 Check
```

```
   0 ALERT level A = Most likely a serious problem - resolve or explain
   1 ALERT level B = A potentially serious problem, consider carefully
  14 ALERT level C = Check. Ensure it is not caused by an omission or oversight
  29 ALERT level G = General information/check it is not something unexpected

   2 ALERT type 1 CIF construction/syntax error, inconsistent or missing data
  21 ALERT type 2 Indicator that the structure model may be wrong or deficient
   9 ALERT type 3 Indicator that the structure quality may be low
   8 ALERT type 4 Improvement, methodology, query or suggestion
   4 ALERT type 5 Informative message, check
```

It is advisable to attempt to resolve as many as possible of the alerts in all categories. Often the minor alerts point to easily fixed oversights, errors and omissions in your CIF or refinement strategy, so attention to these fine details can be worthwhile. It is up to the individual to critically assess their own results and, if necessary, seek expert advice.

PLATON version of 04/06/2025; check.def file version of 30/05/2025

duplicate check

No duplication found

Datablock 2b - ellipsoid plot

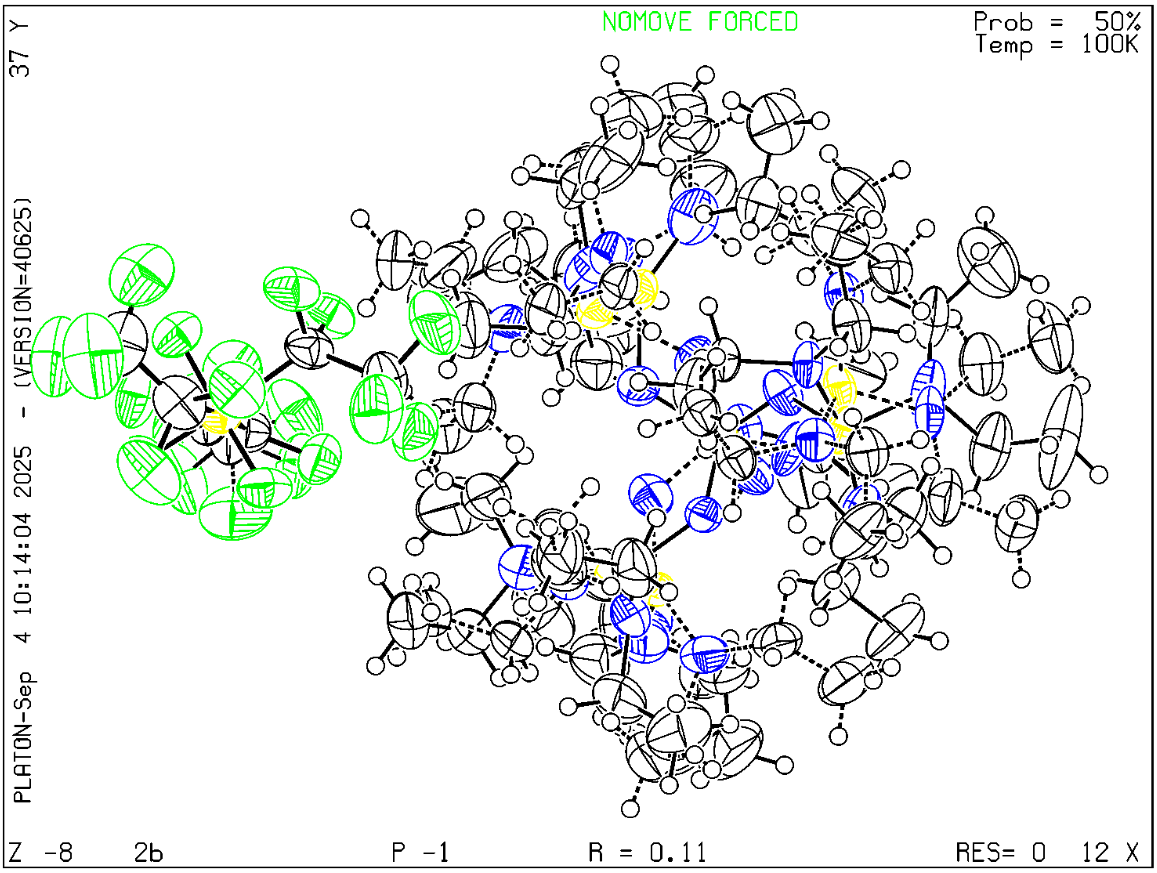

Supplement: Supplementary file 2 — Supporting Information file 2: chem70542‐sup‐0002‐DataFile.zip [file CHEM-32-e03405-s001.zip › checkcif_2b.pdf]
