# Supplementary material for: Oxidative Addition of C‐F Bonds to the Phosphoranide Ion [P(C2F5)2F2]−
Source: Chemistry. 2025 Dec 12;32(2):e03405. doi: 10.1002/chem.202503405 (PMC12790317; doi:10.1002/chem.202503405)

checkCIF/PLATON report

Structure factors have been supplied for datablock(s) 2d

THIS REPORT IS FOR GUIDANCE ONLY. IF USED AS PART OF A REVIEW PROCEDURE FOR PUBLICATION, IT SHOULD NOT REPLACE THE EXPERTISE OF AN EXPERIENCED CRYSTALLOGRAPHIC REFEREE.

No syntax errors found. CIF dictionary Interpreting this report

Datablock: 2d

Bond precision: C-C = 0.0108 A Wavelength=1.54184

Cell:	a=13.1511(6)	b=14.5039(9)	c=17.4474(7)
	alpha=84.574(4)	beta=87.730(4)	gamma=88.303(4)
Temperature:	100 K		

	Calculated	Reported
Volume	3309.3(3)	3309.3(3)
Space group	P -1	P -1
Hall group	-P 1	-P 1
Moiety formula	C40 H99 N13 P4, C7 F18 O P	C40 H99 N13 P4, C7 F18 O P
Sum formula	C47 H99 F18 N13 O P5	C47 H99 F18 N13 O P5
Mr	1359.24	1359.24
Dx,g cm-3	1.364	1.364
Z	2	2
Mu (mm-1)	2.128	2.128
F000	1434.0	1434.0
F000'	1441.94	
h,k,lmax	16,18,21	16,18,21
Nref	13793	13333
Tmin,Tmax		0.659,0.681
Tmin'		

Correction method= # Reported T Limits: Tmin=0.659 Tmax=0.681
AbsCorr = SPHERE

```
Data completeness= 0.967          Theta(max)= 75.883

R(reflections)= 0.1220( 9243)     wR2(reflections)= 0.4073( 13333)

S = 1.564                Npar= 949
```

The following ALERTS were generated. Each ALERT has the format
test-name_ALERT_alert-type_alert-level.
Click on the hyperlinks for more details of the test.

Alert level B

```
PLAT084_ALERT_3_B High wR2 Value (i.e. > 0.25) ..................       0.41 Report
```

Author Response: Due to low quality of the crystal.

```
PLAT340_ALERT_3_B Low Bond Precision on  C-C Bonds ..............    0.01082 Ang.
```

Author Response: Due to low quality of the crystal.

Alert level C

```
DIFMX02_ALERT_1_C  The maximum difference density is > 0.1*ZMAX*0.75
            The relevant atom site should be identified.
```

Author Response: Due to low quality of the crystal.

```
PLAT082_ALERT_2_C High R1 Value .................................       0.12 Report
```

Author Response: Due to low quality of the crystal.

```
PLAT097_ALERT_2_C Large Reported Max.  (Positive) Residual Density       1.46 eA-3
PLAT220_ALERT_2_C NonSolvent   Resd 1  C   Ueq(max)/Ueq(min) Range        3.1 Ratio
PLAT250_ALERT_2_C Large U3/U1 Ratio for <U(i,j)> Tensor(Resd    2)        2.9 Note
PLAT360_ALERT_2_C Short  C(sp3)-C(sp3) Bond  C25      - C26      .       1.41 Ang.
PLAT906_ALERT_3_C Large K Value in the Analysis of Variance ......      5.270 Check
PLAT911_ALERT_3_C Missing FCF Refl Between Thmin & STh/L=    0.600         90 Report
               -6  1  0,  -4  1  0,  -5  3  0,  -2  3  0,  -1  3  0,  -5 15  0,
                0 17  0,   2 17  0,  -8 -2  1,  -6 -1  1,   4 -1  1,   1  1  1,
               12  1  1,  -4  3  1,  -4 -4  2,  -5 -2  2,  -4 -1  2,   5 -1  2,
                0  0  2,  -6  1  2,   6  1  2,   0  5  2,  -4 14  2,   0 17  2,
              -10 -4  3,  -6 -1  3,  -5 -1  3,   1 -1  3,   7 -1  3,  -4  2  3,
                   (   60 More Missing: see the .ckf listing file)
PLAT934_ALERT_3_C Number of (Iobs-Icalc)/Sigma(W) > 10 Outliers ..          1 Check
                2 -2  1,
PLAT971_ALERT_2_C Check Calcd Resid. Dens.  0.14Ang From N2A             1.52 eA-3
PLAT977_ALERT_2_C Check Negative Difference Density on H26A      .      -0.31 eA-3
PLAT977_ALERT_2_C Check Negative Difference Density on H37B      .      -0.37 eA-3
```

```
PLAT977_ALERT_2_C Check Negative Difference Density on H37C      .       -0.41 eA-3
```

Alert level G

```
PLAT002_ALERT_2_G Number of Distance or Angle Restraints on AtSite          9 Note
PLAT003_ALERT_2_G Number of Uiso or U(i,j)  Restrained non-H-Atoms         76 Report
PLAT072_ALERT_2_G SHELXL First  Parameter in WGHT  Unusually Large       0.20 Report
PLAT154_ALERT_1_G The s.u.'s on the Cell Angles are Equal ..(Note)      0.004 Degree
PLAT171_ALERT_4_G The CIF-Embedded .res File Contains EADP Records          4 Report
PLAT176_ALERT_4_G The CIF-Embedded .res File Contains SADI Records          2 Report
PLAT178_ALERT_4_G The CIF-Embedded .res File Contains SIMU Records          6 Report
PLAT187_ALERT_4_G The CIF-Embedded .res File Contains RIGU Records          6 Report
PLAT188_ALERT_3_G A Non-default SIMU Restraint Value has been used     0.0010 Report
PLAT188_ALERT_3_G A Non-default SIMU Restraint Value has been used     0.0100 Report
PLAT188_ALERT_3_G A Non-default SIMU Restraint Value has been used     0.0100 Report
PLAT188_ALERT_3_G A Non-default SIMU Restraint Value has been used     0.0100 Report
PLAT188_ALERT_3_G A Non-default SIMU Restraint Value has been used     0.0100 Report
PLAT188_ALERT_3_G A Non-default SIMU Restraint Value has been used     0.0100 Report
PLAT190_ALERT_3_G A Non-default RIGU Restraint Value for First Par     0.0010 Report
PLAT190_ALERT_3_G A Non-default RIGU Restraint Value for SecondPar     0.0010 Report
PLAT190_ALERT_3_G A Non-default RIGU Restraint Value for First Par     0.0100 Report
PLAT190_ALERT_3_G A Non-default RIGU Restraint Value for SecondPar     0.0100 Report
PLAT190_ALERT_3_G A Non-default RIGU Restraint Value for First Par     0.0100 Report
PLAT190_ALERT_3_G A Non-default RIGU Restraint Value for SecondPar     0.0100 Report
PLAT190_ALERT_3_G A Non-default RIGU Restraint Value for First Par     0.0100 Report
PLAT190_ALERT_3_G A Non-default RIGU Restraint Value for SecondPar     0.0100 Report
PLAT190_ALERT_3_G A Non-default RIGU Restraint Value for First Par     0.0100 Report
PLAT190_ALERT_3_G A Non-default RIGU Restraint Value for SecondPar     0.0100 Report
PLAT190_ALERT_3_G A Non-default RIGU Restraint Value for First Par     0.0100 Report
PLAT190_ALERT_3_G A Non-default RIGU Restraint Value for SecondPar     0.0100 Report
PLAT230_ALERT_2_G Hirshfeld Test Diff for    P2A      --N7       .        9.3 s.u.
PLAT242_ALERT_2_G Low    'MainMol' Ueq as Compared to Neighbors of        C42 Check
PLAT242_ALERT_2_G Low    'MainMol' Ueq as Compared to Neighbors of        C44 Check
PLAT242_ALERT_2_G Low    'MainMol' Ueq as Compared to Neighbors of        C47 Check
PLAT299_ALERT_4_G Atom Site Occupancy Constrained at ............         0.5 Check
              P2A     P2B     P4A     P4B     N2A     N2B     N4A     N4B
              N5A     N5B     N6A     N6B     N12A    N12B    N13A    N13B
              C5A     C5B     C6A     C6B     C7A     C7B     C8A     C8B
              C9A     C9B     C10A    C10B    C11A    C11B    C12A    C12B
              C33A    C33B    C34A    C34B    C35A    C35B    C36A    C36B
              C37A    C37B    C38A    C38B    C39A    C39B    C40A    C40B
              H5AA    H5AB    H5BA    H5BB    H6AA    H6AB    H6AC    H6BA
              H6BB    H6BC    H10A    H10B    H10C    H10D    H10E    H10F
              H7AA    H11A    H11B    H11C    H11D    H7AB    H12A    H12B
              H12C    H12D    H12E    H12F    H7BA    H7BB    H8AA    H8AB
              H8AC    H8BA    H8BB    H8BC    H9AA    H9AB    H9BA    H9BB
              H33A    H33B    H33C    H33D    H34A    H34B    H34C    H34D
              H34E    H34F    H35A    H35B    H35C    H35D    H36A    H36B
              H36C    H36D    H36E    H36F    H37A    H37B    H37C    H37D
              H38A    H38B    H38C    H38D    H38E    H38F    H39A    H39B
              H39C    H39D    H40A    H40B    H40C    H40D    H40E    H40F
PLAT301_ALERT_3_G Main Residue  Disorder .............(Resd    1)         42% Note
PLAT410_ALERT_2_G Short Intra H...H Contact  H31A     ..H37A     .       2.04 Ang.
                                                      x,y,z  =      1_555 Check
PLAT410_ALERT_2_G Short Intra H...H Contact  H31B     ..H37A     .       2.03 Ang.
                                                      x,y,z  =      1_555 Check
PLAT412_ALERT_2_G Short Intra XH3 .. XHn     H6BC     ..H14C     .       1.73 Ang.
```

```
                                                     x,y,z  =      1_555 Check
PLAT412_ALERT_2_G Short Intra XH3 .. XHn     H30C     ..H38C     .       2.12 Ang.
                                                     x,y,z  =      1_555 Check
PLAT412_ALERT_2_G Short Intra XH3 .. XHn     H32C     ..H34F     .       2.10 Ang.
                                                     x,y,z  =      1_555 Check
PLAT413_ALERT_2_G Short Inter XH3 .. XHn     H6AC     ..H26B     .       1.88 Ang.
                                                   x,1+y,z  =      1_565 Check
PLAT432_ALERT_2_G Short Inter X...Y Contact  F10      ..C40B     .       2.83 Ang.
                                                1+x,-1+y,z  =      1_645 Check
PLAT432_ALERT_2_G Short Inter X...Y Contact  C6A      ..C26      .       2.81 Ang.
                                                   x,1+y,z  =      1_565 Check
PLAT720_ALERT_4_G Number of Unusual/Non-Standard Labels ..........         24 Note
              H5AA    H5AB    H5BA    H5BB    H6AA    H6AB    H6AC    H6BA
              H6BB    H6BC    H7AA    H7AB    H7BA    H7BB    H8AA    H8AB
              H8AC    H8BA    H8BB    H8BC    H9AA    H9AB    H9BA    H9BB
PLAT811_ALERT_5_G No ADDSYM Analysis: Too Many Excluded Atoms ....          ! Info
PLAT860_ALERT_3_G Number of Least-Squares Restraints ............        897 Note
PLAT910_ALERT_3_G Missing FCF Reflection(s) Below Theta(Min)[Deg]=       3.06 Note
                0  0  1,
PLAT912_ALERT_4_G Missing # of FCF Reflections Above STh/L=  0.600        369 Note
PLAT913_ALERT_3_G Missing # of Very Strong Reflections in FCF ....          1 Note
                0  0  2,
PLAT941_ALERT_3_G Average HKL Measurement Multiplicity ..........         2.1 Low
PLAT969_ALERT_5_G The 'Henn et al.' R-Factor-gap value ..........       6.702 Note
              Predicted wR2: Based on SigI**2  6.08 or SHELX Weight 26.04
PLAT978_ALERT_2_G Number C-C Bonds with Positive Residual Density.          0 Info
PLAT992_ALERT_5_G Repd & Actual _reflns_number_gt Values Differ by          2 Check
```

```
  0 ALERT level A = Most likely a serious problem - resolve or explain
  2 ALERT level B = A potentially serious problem, consider carefully
 13 ALERT level C = Check. Ensure it is not caused by an omission or oversight
 50 ALERT level G = General information/check it is not something unexpected

  2 ALERT type 1 CIF construction/syntax error, inconsistent or missing data
 25 ALERT type 2 Indicator that the structure model may be wrong or deficient
 28 ALERT type 3 Indicator that the structure quality may be low
  7 ALERT type 4 Improvement, methodology, query or suggestion
  3 ALERT type 5 Informative message, check
```

It is advisable to attempt to resolve as many as possible of the alerts in all categories. Often the minor alerts point to easily fixed oversights, errors and omissions in your CIF or refinement strategy, so attention to these fine details can be worthwhile. It is up to the individual to critically assess their own results and, if necessary, seek expert advice.

PLATON version of 04/06/2025; check.def file version of 30/05/2025

---

duplicate check

No duplication found

Datablock 2d - ellipsoid plot

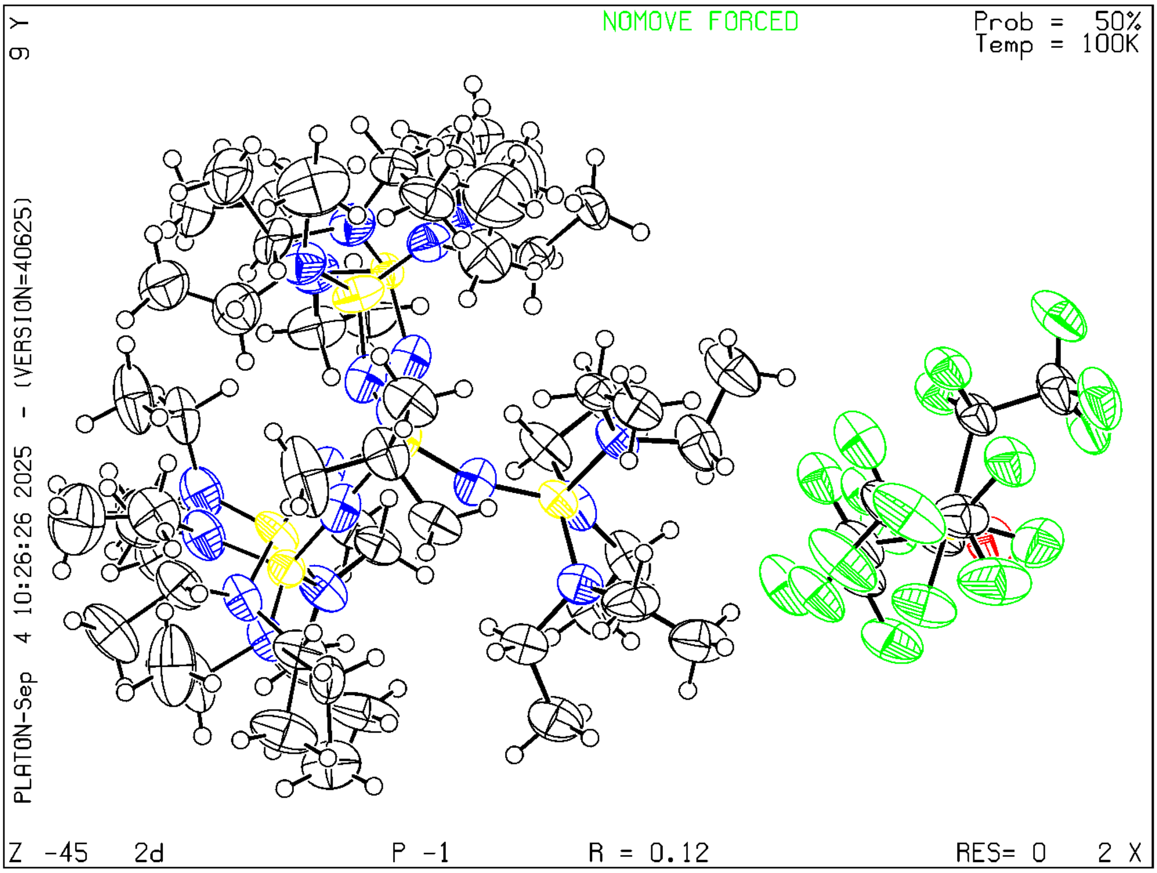

Supplement: Supplementary file 2 — Supporting Information file 2: chem70542‐sup‐0002‐DataFile.zip [file CHEM-32-e03405-s001.zip › checkcif_2d.pdf]
